# Fibrogenesis Biomarkers and Clinical Disease Progression in ADPKD

**DOI:** 10.1016/j.ekir.2026.107102

**Published:** 2026-09-23

**Authors:** Solveig Skovlund Groen, Ron T. Gansevoort, Nadja Sparding, Judith E. Heida, Esther Meijer, Morten A. Karsdal, Peder Frederiksen, Federica Genovese, Thomas Bais

**Affiliations:** 1 Cardiovascular & Renal Research, Nordic Bioscience, Herlev, Denmark; 2 Division of Nephrology, Department of Internal Medicine, University Medical Center Groningen, University of Groningen, Groningen, The Netherlands

**Keywords:** ADPKD, biomarker, collagen, extracellular matrix, fibrosis

## Abstract

**Introduction:**

Fibrogenesis contributes to disease progression in patients with autosomal dominant polycystic kidney disease (ADPKD). In this exploratory study, we evaluated the association of protease-generated circulating and urinary collagen fragments (type III collagen degradation [C3M], type III collagen formation [PRO-C3], and type VI collagen formation [PRO-C6]), reflecting fibrogenesis and fibrolysis, with clinical outcomes in ADPKD.

**Methods:**

We included 302 patients from the DIPAK-1 trial (lanreotide vs. standard treatment), with a mean baseline age of 48.9 ± 7.3 years and estimated glomerular filtration rate (eGFR) of 51.3 ± 11.5 ml/min per 1.73 m^2^. Kidney endpoints were eGFR slope, rapid disease progression (eGFR loss ≥3 ml/min per 1.73 m^2^ per year), and a composite kidney endpoint (30% eGFR loss or initiation of kidney replacement therapy). Net benefit analysis was performed to assess clinical utility.

**Results:**

During 2.4 ± 0.4 years of follow-up, mean eGFR slope was −4.02 ± 3.64 ml/min per 1.73 m^2^ per year, 190 patients (62.9%) had rapid disease progression, and 87 (28.8%) reached the composite endpoint. After adjustment for sex, baseline age, baseline eGFR, Mayo imaging class (MIC), *PKD* pathogenic variant and treatment group, uPRO-C3/Cr and uPRO-C6/Cr were independently associated with eGFR slope, rapid disease progression, and the composite endpoint. uPRO-C3/Cr was also associated with HtTKV growth rate. Adding uPRO-C3/Cr or uPRO-C6/Cr improved prediction of rapid disease progression, increasing area under curve (AUC) from 0.78 (95% confidence interval [CI], 0.73–0.86) to 0.79 (95% CI, 0.73–0.86) and 0.80 (95% CI, 0.73–0.86), respectively. Decision curve analysis did not show a meaningful net benefit.

**Conclusion:**

These findings demonstrate that uPRO-C3/Cr and uPRO-C6/Cr were independently associated with kidney outcomes in ADPKD, providing clinical evidence that extracellular matrix (ECM) remodeling is associated with ADPKD disease progression.

## Graphical abstract

**Figure:**
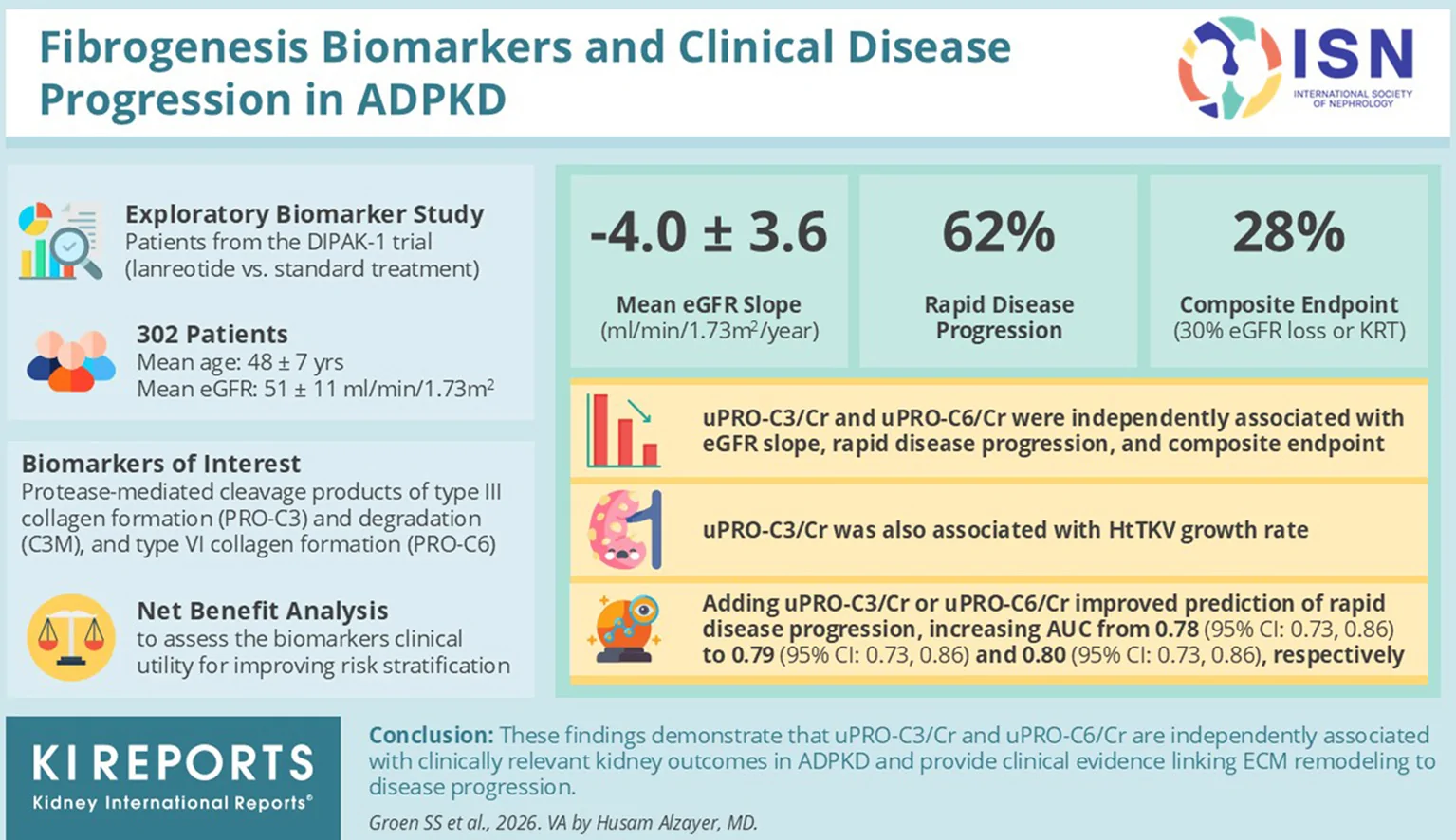

ADPKD is the leading genetic cause of kidney failure, and is characterized by progressive kidney cyst growth and gradual loss of kidney function.^1^ The rate of disease progression and age at onset of kidney failure vary widely among patients with ADPKD. Predicting disease progression and kidney failure remains challenging, but it is important for counseling, treatment decisions, and selecting patients for clinical trials.

Most patients with ADPKD carry pathogenic *PKD1* or *PKD2* variants, which encode polycystin-1 and -2, respectively. These proteins form a complex located on the primary cilia of renal tubular cells, acting as a nonselective cation channel permeable to Ca^2+^. Abnormal polycystin signaling in these cells disrupts intracellular Ca^2+^ homeostasis, increases cyclic adenosine monophosphate concentrations, and promotes cystogenesis through activation of proliferative signaling pathways.^2^ The expansion of kidney cysts imposes mechanical stress on the adjacent parenchyma, which promotes inflammation and fibrosis in the renal interstitium, thereby accelerating nephron loss and kidney function decline.^3^ In histopathologic samples of patients with ADPKD, tubulointerstitial fibrosis is associated with clinical disease progression, and development of kidney failure and can be found even in early disease phases.^4^

Tubulointerstitial fibrosis is observed in many forms of chronic kidney disease (CKD) and presents a final common pathway that contributes to the development of kidney failure. It is characterized by a persistent inflammatory infiltrate, an increase in interstitial cells with appearance of myofibroblasts, epithelial-to-mesenchymal transition, tubular atrophy, and accumulation of ECM components.^3^ Similar pathophysiologic changes are observed in ADPKD. In polycystic kidneys, injured tubular epithelial cells release proinflammatory cytokines like transforming growth factor-β, which drives tubular cell dedifferentation, recruitment of inflammatory cells, and activation of myofibroblasts. Activated myofibroblasts deposit ECM components like fibrillar type III collagen and beaded filament type VI collagen, which are cleaved by matrix metalloproteinases (MMPs) during maturation.^5^ This releases protease-mediated cleavage products of PRO-C3, C3M, and PRO-C6 into the circulation and urine (Figure 1). Therefore, these peptides may serve as biomarkers that reflect ongoing ECM remodeling and fibrogenesis in polycystic kidneys.

**Figure 1:**
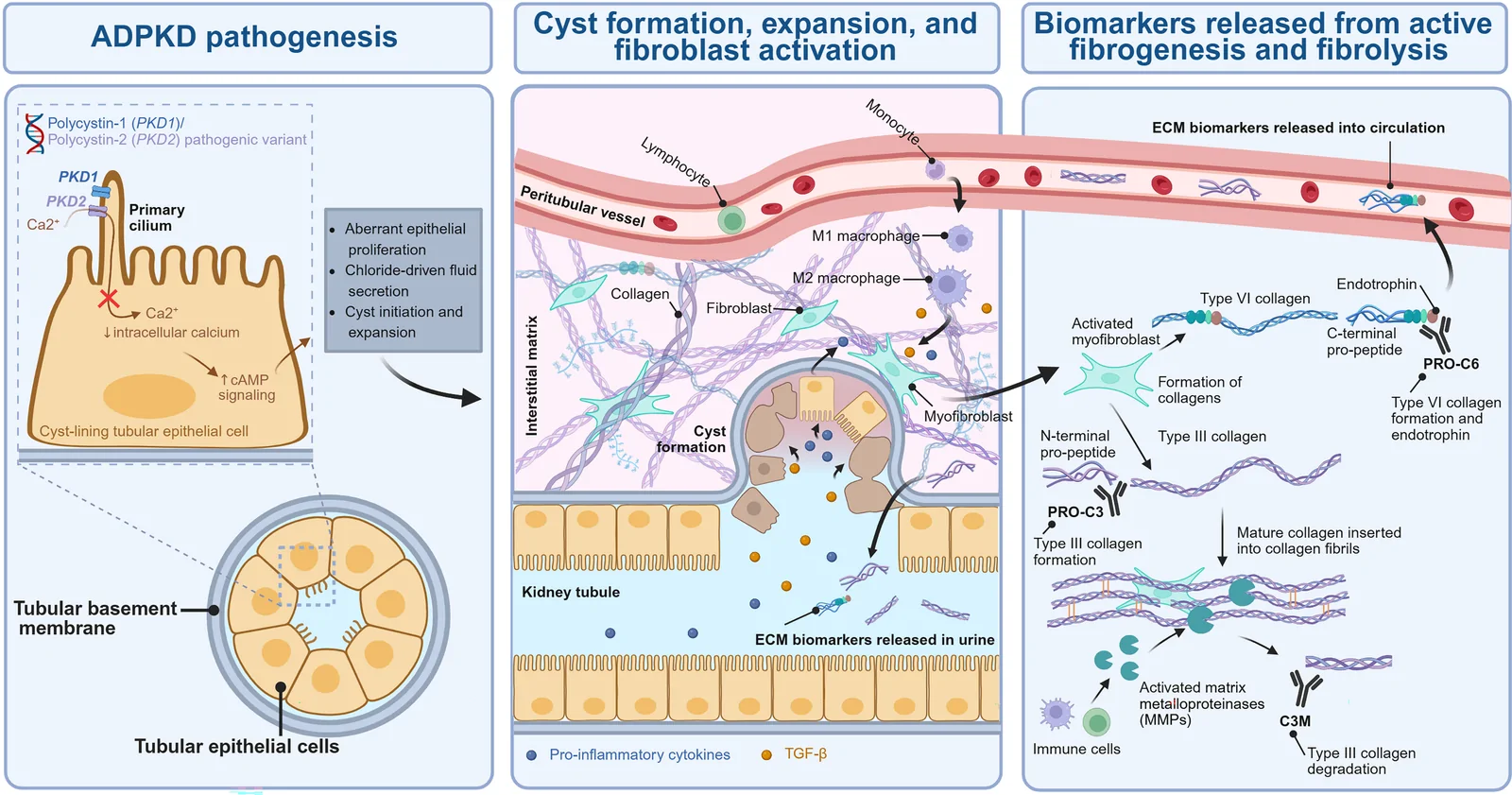
Mechanistic biomarkers released from extracellular matrix fibrogenesis and fibrolysis during ADPKD progression. Pathogenic variants in *PKD1* and *PKD2* lead to cyst formation and expansion into the kidney interstitium. This activates injury-related pathways in which tubular epithelial cells release proinflammatory cytokines, including TGF-β, promoting tubular cell dedifferentiation and recruitment of inflammatory cells such as macrophages. M2-like macrophages accumulate around cysts and secrete inflammatory and profibrotic mediators that stimulate tubular cell proliferation and myofibroblast activation. Activated myofibroblasts synthesize and deposit extracellular matrix components, leading to matrix accumulation and fibrosis. During fibrogenesis of fibrillar type III collagen and beaded filament type VI collagen, propeptides are released from the mature collagen before insertion into collagen fibrils. During fibrolysis, collagens are processed by matrix metalloproteinases, generating neo-epitope fragments that, like the propeptides, can be detected in circulation and urine. These propeptide and neo-epitope fragments may serve as biomarkers reflecting type III collagen formation (PRO-C3), type III collagen degradation (C3M), and type VI collagen formation (PRO-C6). ADPKD, autosomal dominant polycystic kidney disease; TGF-β, transforming growth factor.

In this exploratory study, we investigated whether PRO-C3, C3M, and PRO-C6 in serum and urine are associated with kidney endpoints in patients with ADPKD.

## Methods

### Participants and Procedures

Patients with ADPKD from an open-label randomized clinical trial (DIPAK-1)^6^ with blinded endpoint assessment were included for this exploratory biomarker study. The DIPAK-1 study investigated the effect of the somatostatin analogue lanreotide on kidney function decline and kidney growth. The main findings were that lanreotide did not reduce the rate of kidney function decline, but significantly decreased kidney volume growth.^6^ The patients were recruited from July 2012 to March 2015 at 4 nephrology outpatient clinics in the Netherlands. Follow-up visits of the 2.5-year trial ended in August 2017. ADPKD was diagnosed based on the modified Ravine criteria.^7^ Patients were between 18 and 60 years of age and had an eGFR of 30 to 60 ml/min per 1.73 m^2^. The main exclusion criteria were a history of gallstones or pancreatitis, and diseases or use of medications that influence kidney function, like diabetes mellitus, chronic NSAID use, or use of tolvaptan. Patients were randomized 1:1 to receive either lanreotide (120 mg subcutaneously once every 4 weeks) in addition to standard care, or standard care only. Standard care consisted of adequate blood pressure measurement (target < 140/90 mm Hg), a sodium-restricted diet and preferably a first-choice blood pressure-lowering agent (angiotensin-converting enzyme inhibitor or angiotensin receptor blocker). The use of tolvaptan was not permitted during the study. Of the 305 patients included in the primary DIPAK-1 analysis, 3 patients did not have serum or urinary samples available, leaving 302 patients for the analysis. The study adhered to the International Conference on Harmonization Good Clinical Practice guidelines and was approved by the institutional review board of the University Medical Center Groningen (code: 042). Written informed consent was obtained from all participants.

### Clinical and Laboratory Measurements

Fasting blood samples were drawn for the measurement of serum creatinine and cystatin C, and *PKD* pathogenic variant analyses. Serum creatinine was measured using an isotope-dilution mass spectrometry-traceable enzymatic method, and cystatin C using reference material from the International Federation of Clinical Chemistry. Samples from the same patient were measured in 1 run to minimize interassay variation. eGFR_Cr_ values were calculated using the 2009 creatinine-based CKD Epidemiology Collaboration equation,^8^ which reflects present clinical practice across the Europe.^9^ For the sensitivity analysis, eGFR_cys_ values were calculated using the 2012 cystatin C-based CKD Epidemiology Collaboration formula.^10^ The *PKD* pathogenic variant analysis was performed with DNA isolation using PUREGENE nucleic acid purification chemistry on the AUTOPURE LS 98 platform (Qiagen, Venlo, the Netherlands), followed by sequencing of amplified coding exons directly (exons 34–46) or sequencing of long-range polymerase chain reaction products (exons 1–33). Blood pressure was calculated as the average of 3 automated measurements. Magnetic resonance imaging was performed to measure total kidney volume (TKV) using a standardized abdominal magnetic resonance imaging protocol without contrast. TKV was measured on T2-weighted coronal images using Analyze 9.0 (AnalyzeDirect Inc., Overland Park, KS). Height-adjusted TKV (HtTKV) was calculated by dividing TKV by height and indexed for age to classify patients according to Mayo Imaging Class (MIC).^11^ The day before the baseline visit, patients collected a 24-hour urine sample which was biobanked and stored at −80°C. The methodology of the DIPAK-1 study was previously reported.^12^ Urinary albumin and creatinine were measured using standard laboratory techniques, and urinary monocyte chemoattractant protein (MCP-1) was measured using enzyme-linked immunosorbent assays (ELISA; R&D Systems Inc., Minneapolis, MN), with a lower detection limit of 0.04 ng/ml.

### Measurements of ECM Biomarkers in Serum and Urine

The ECM biomarkers C3M (nordicC3M), PRO-C3 (nordicPRO-C3), and PRO-C6 (nordicPRO-C6) were selected for measurement based on assay reliability and the pathophysiological role of these peptides in kidney fibrosis. These biomarkers were initially quantified at baseline in serum (sC3M, sPRO-C3, and sPRO-C6) and 24-hour urine samples (uC3M, uPRO-C3, and uPRO-C6). Furthermore, at week 12, 120, and 132 follow-up visits, all biomarkers were measured in serum and urine except for serum PRO-C3 (sPRO-C3). The decision to exclude PRO-C3 was based on the initial screening phase, where only biomarkers showing an association with disease progression were selected for repeated measurements in the full cohort. The biomarkers were measured using competitive ELISAs (Nordic Bioscience, Herlev, Denmark) according to the manufacturer’s instructions, as detailed in the Supplementary References in Supplementary Table S1. Both the serum and urine C3M assays reflect type III collagen degradation but use different ELISA settings and monoclonal antibody clones to detect the sequence KNGETGPQGP, corresponding to the cleavage site of MMP-9 in position 610 of type III collagen.^13^ The PRO-C3 assay reflects type III collagen formation and uses a monoclonal antibody detecting the sequence CPTGPQNYSP corresponding to the cleavage site of the N-terminal propeptide from the mature collagen in position 153 of the type III collagen α1 chain.^14^ The PRO-C6 assay targets the C-terminal portion of the α3 chain (sequence KPGVISVMGT) of type VI collagen, which reflects collagen formation and the release of the bioactive matrikine endotrophin.^15^ Assays were accepted with an intraassay coefficient of variation ≤ 10% and an interassay coefficient of variation ≤ 15%. Personnel who performed the biomarker measurements were blinded to the clinical study data. Serum and urine levels of biomarkers were used to calculate fractional excretions. Urinary ECM biomarker concentrations were normalized for urine creatinine (uC3M/Cr, uPRO-C3/Cr, and uPRO-C6/Cr) to account for urinary concentration effects. Urinary creatinine was measured by the QuantiChrom creatinine assay kit (BioAssay system, Hayward, CA).

### Statistical Analysis

Statistical analysis was performed using R version 4.3.2 (R Foundation for Statistical Computing, Vienna, Austria)^16^ and RStudio version 2022.12.0+353 (Posit Software, PBC).^17^ Continuous variables and biomarkers were transformed using the natural logarithm if needed and z-score was standardized. Multivariable models were adjusted for sex, age, baseline eGFR, MIC, *PKD* pathogenic variant type (*PKD1* truncating, *PKD1* nontruncating, and *PKD2*), and treatment group. The MIC and pathogenic variant type are established predictors of ADPKD disease progression.^11^^,^^18^

The effect of baseline biomarker levels on baseline eGFR (cross-sectional analysis) was performed using linear multivariable regression. To investigate the effects on annual rate of eGFR decline (eGFR slope), linear mixed effects models for repeated measures were used with random effects for time and subjects and an unstructured covariance structure. Fixed effects were modeled as interactions with time to estimate their effect on eGFR slope. A similar approach was used to investigate the effects on HtTKV growth rate. For this exploratory outcome, HtTKV was log-transformed, and the effect estimates were then back-transformed to express the effects on HtTKV growth rate (% per year).

Rapid disease progression was defined as an eGFR loss of ≥ 3.0 ml/min per 1.73 m^2^ per year. This cut-off value was chosen for its clinical relevance because European guidelines recommend using it to assess when to start disease-modifying treatments like tolvaptan.^19^ Multivariable logistic regression models were used to investigate the effect of biomarker levels on the odds of rapid disease progression. Predicted probabilities of logistic regression models were assessed based on receiver operating characteristic curve analysis and area under curve (AUC) values. In addition, we evaluated the incremental clinical utility by calculating net benefit across a clinically plausible range of decision thresholds (0.1–0.5), comparing the biomarker model to the baseline model at each threshold. Net benefit incorporates the trade-off between true positives and false positives, with false positives weighted according to the chosen threshold probability. Decision thresholds represent cut-offs applied to model-predicted probabilities to classify individuals as positive or negative; lower thresholds (e.g., 0.1) favor higher sensitivity at the expense of more false positives, whereas higher thresholds (e.g., 0.5) favor higher specificity. Net benefit is expressed per patient and represents the number of net true positives per patient after accounting for false positives using the threshold-specific weighting (and can be scaled to per 100 patients by multiplying by 100). For example, a net benefit of 0.03 corresponds to a net gain of approximately 3 true-positive classifications per 100 patients at the specified decision threshold. AUC and net benefit values were bootstrapped with 1000 iterations to obtain the median value along with 95% CI. Nested logistic regression models were compared using the likelihood ratio test.

Kidney failure was defined as the initiation of kidney replacement therapy (dialysis or kidney transplantation) or developing an eGFR ≤15 ml/min per 1.73 m^2^, whichever occurred first. The primary outcome for survival analysis was a composite endpoint of 30% decline in eGFR from baseline or kidney failure. In addition to Kaplan-Meier estimates, multivariable Cox regression survival analysis was performed for the composite endpoint. C-indices were computed for the Cox regression models and bootstrapped with 1000 iterations to derive 95% CI. The likelihood ratio test was used to compare nested Cox regression models.

Along with baseline biomarker levels, repeated follow-up measurements (week 12, 120, and 132) were used to evaluate the correlation between biomarker slope, eGFR slope, and HtTKV growth rate.

### Sensitivity and Subgroup Analyses

The analyses for urinary biomarkers were repeated using 24-hour urinary excretions. In addition, the main analyses were repeated with cystatin C-based eGFR values. The analyses were also repeated with correction for urinary albumin/creatinine (uACR) and urinary MCP-1/creatinine (uMCP-1/Cr) ratio. These biomarkers were strongly associated with disease progression in ADPKD according to a previous study.^20^ To compare the performance of ECM biomarkers with MCP-1, we also evaluated the addition of MCP-1 to logistic regression models predicting rapidly progressive disease, allowing comparison of the increase in AUC.

To account for missing data, the analyses were repeated with imputation for missing biomarker and covariate data. Multiple data imputation (*m* = 5) was performed using the mice package.^21^ Imputations were based on all variables included in the model and generated by predictive mean matching. Parameter estimates were pooled by Rubin’s method after fitting each imputed dataset to the models.

We repeated the main analyses in patients with MIC 1C. These patients have intermediate rates of disease progression, and guidelines recommend obtaining additional evidence of rapid disease progression before initiating tolvaptan treatment. Therefore, these patients are likely to benefit most from the incorporation of biomarkers in risk stratification.

## Results

### Cohort Characteristics

A total of 302 patients with a mean age of 48.9 ± 7.3 years and a mean eGFR of 51.3 ± 11.5 ml/min per 1.73 m^2^ at baseline were included in the analyses. Of note, 1 patient died during the study from squamous cell lung carcinoma. Baseline characteristics are summarized in Table 1. Nearly half of patients were male (46.4%), and most were Caucasian (97.4%). Most patients (78.1%) had relatively high-risk MIC (1C/1D/1E). The most prevalent pathogenic variant type was *PKD1* truncating (46.0%), followed by *PKD1* nontruncating (24.2%) and *PKD2* variants (19.2%).

**Table 1:** Baseline characteristics of the DIPAK-1 cohort

Variable	Overall (*N* = 302)
Male sex	140 (46.4)
Age at baseline, yr	48.9 ± 7.3
Ethnicity	
-Asian	3 (1.0)
-Caucasian	294 (97.4)
-Other	5 (1.7)
BMI, kg/m^2^	27.0 ± 4.7
Systolic blood pressure, mm Hg	132.9 ± 13.2
Mayo Imaging Class	
-1A/1B	49 (16.2)
-1C	107 (35.4)
-1D/1E	129 (42.7)
-Atypical	11 (3.6)
-Not available	6 (2.0)
Pathogenic variant type	
-*PKD2*	58 (19.2)
-*PKD1* nontruncating	73 (24.2)
-*PKD1* truncating	139 (46.0)
-Not available	32 (10.6)
eGFR, ml/min per 1.73 m^2^	51.3 ± 11.5
sC3M, ng/ml	16.5 (5.6)
sPRO-C3, ng/ml	12.8 (5.3)
sPRO-C6, ng/ml	11.3 (4.3)
uC3M/Cr, ng/mg	39.7 (18.1)
uPRO-C3/Cr, ng/mg	6.4 (4.6)
uPRO-C6/Cr, ng/mg	2.1 (1.2)

Data are given as number (with percentage), mean ± SD, or median [interquartile range]. BMI, body mass index.

The average follow-up duration was 2.4 ± 0.4 years, and the median number of eGFR measurements was 9.^8^^,^^13^ Disease progression parameters are displayed in Supplementary Table S2. Between the lanreotide and standard care treatment arms, there were no differences between eGFR slope, incidence of rapid disease progression, or reaching the composite kidney endpoint, as previously reported by Meijer *et al*.^6^ Baseline characteristics with stratification per tertile of uPRO-C3/Cr and uPRO-C6/Cr are displayed in Supplementary Tables S3 and S4, respectively. For both uPRO-C3/Cr and uPRO-C6/Cr, patients in higher tertiles were more often female (*P* < 0.001 and *P* = 0.002, respectively). In addition, patients in higher uPRO-C3/Cr tertiles had lower eGFR at baseline (*P* = 0.005).

At baseline, sPRO-C3 and sPRO-C6 levels were available for all 302 patients, and sC3M for 252/302 (83.4%) patients. Urine biomarkers (uC3M/Cr, uPRO-C3/Cr, and uPRO-C6/Cr) were measured for 222/302 patients (73.5%) that had available urine samples. Baseline characteristics were similar between patients with and without available urinary biomarker data (Supplementary Table S5), suggesting that missing urinary biomarker data were unlikely to be systematically different from nonmissing data.

### Biomarker Measurements at Baseline

The correlations between biomarker levels transformed using the natural logarithm at baseline are shown in Supplementary Figure S1. Moderate to strong correlations were present between sPRO-C3 and sPRO-C6 (*R* = 0.457; *P* < 0.001), and uPRO-C3/Cr and uPRO-C6/Cr (*R* = 0.642; *P* < 0.001). Interestingly, the correlations between sPRO-C3 and sPRO-C6 and their urinary counterparts (uPRO-C3/Cr and uPRO-C6/Cr) were weak. The remaining fibrogenesis biomarkers were weakly or not correlated. sC3M, uPRO-C3/Cr, and uPRO-C6/Cr were significantly correlated with uACR levels (all *P* < 0.001), although the Pearson *R* correlations were relatively weak (*R =* 0.226–0.276), as displayed in Supplementary Figure S1.

The mean ± SD fractional excretions for C3M, PRO-C3, and PRO-C6 were 3.5 ± 2.1, 1.0 ± 1.1 and 0.4 ± 0.5%, respectively, which were significantly higher than for albumin (0.002 ± 0.004%, *P* < 0.001 for all 3 comparisons).

### Association With Baseline eGFR and Baseline HtTKV

The associations of baseline biomarker levels with baseline eGFR and baseline HtTKV are shown in Supplementary Table S7. After correction for sex, baseline age, baseline eGFR (except when this was the dependent variable), MIC, *PKD* pathogenic variant type, and treatment group, most biomarkers were significantly associated with baseline eGFR. In contrast, none of the biomarkers were associated with baseline HtTKV.

### Associations With eGFR Slope

The associations of baseline biomarker levels with eGFR slope are displayed in Table 2. After correction for covariates, baseline levels of sC3M, uPRO-C3/Cr, and uPRO-C6/Cr were independently associated with eGFR slope. This association was strongest between uPRO-C6/Cr and eGFR slope, with a standardized β of −0.74 (95% CI, −1.08 to −0.39; *P* < 0.001). Linear mixed effects models for the effects on eGFR slope with correction for an increasing number of covariates are displayed in Supplementary Table S8.

**Table 2:** Summary of predictive models that include baseline biomarker levels to predict kidney function endpoints

	eGFR slope		Rapidly progressive disease		Composite kidney endpoint		HtTKV growth rate	
Variable	Standardized β (95% CI)	*P*-value	OR (95% CI)	*P*-value	HR (95% CI)	*P*-value	Standardized β (95% CI)	*P*-value
Serum								
sC3M	−0.35 (−0.68 to −0.01)	0.04	1.01 (0.7, 1.39)	0.94	1.20 (0.9, 1.51)	0.14	0.25 (−0.39 to 0.88)	0.45
sPRO−C3	0.25 (−0.05 to 0.55)	0.11	0.77 (0.5, 1.02)	0.07	0.95 (0.7, 1.18)	0.64	0.07 (−0.51 to 0.65)	0.81
sPRO-C6	−0.25 (−0.63 to 0.14)	0.21	0.84 (0.6, 1.19)	0.34	1.18 (0.8, 1.59)	0.26	0.31 (−0.41 to 1.03)	0.41
Urine								
uC3M/Cr	0.12 (−0.29 to 0.53)	0.57	1.14 (0.7, 1.69)	0.53	0.78 (0.5, 1.08)	0.14	−0.18 (−0.98 to 0.63)	0.66
uPRO-C3/Cr	−0.56 (−0.92 to −0.21)	0.002	1.46 (1.0, 2.14)	0.04	1.45 (1.1, 1.83)	0.001	0.75 (0.06, 1.45)	0.04
uPRO-C6/Cr	−0.74 (−1.08 to −0.39)	< 0.001	1.92 (1.2, 3.13)	0.005	1.38 (1.1, 1.72)	0.003	0.04 (−0.66 to 0.74)	0.91

CI, confidence interval; eGFR, estimated glomerular filtration rate; HR, hazard ratio; HtTKV, height-adjusted total kidney volume; OR, odds ratio.

Kidney endpoints included the eGFR slope, development of rapidly progressive disease, reaching the composite kidney endpoint and HtTKV growth rate. All models were corrected for sex, baseline age, baseline eGFR, MIC, *PKD* pathogenic variant type, and treatment group. One biomarker was included per model. Biomarkers were transformed using the natural logarithm and z-score was standardized.

### Associations With Rapid Disease Progression

In total, 190/302 (62.9%) of patients exhibited rapidly progressive disease. After correction for covariates, baseline uPRO-C3/Cr (*P* = 0.04) and uPRO-C6/Cr levels (*P* = 0.005) were independently associated with rapid disease progression (Table 2). Of these, the association between rapidly progressive disease and uPRO-C6/Cr appeared stronger than with uPRO-C3/Cr (adjusted odds ratio, 1.92 [95% CI, 1.25–3.13] and 1.46 [95% CI, 1.02–2.14], respectively). Logistic regression models corrected for an increasing number of covariates are summarized in Supplementary Table S9.

Receiver operating characteristic curves of logistic regression models that predict rapidly progressive disease are presented in Figure 2. After bootstrapping, the median AUC value of a baseline model combining sex, baseline age, baseline eGFR, MIC, *PKD* pathogenic variant type, and treatment group, was 0.78 (95% CI, 0.71–0.84). The addition of uPRO-C3/Cr to the baseline model slightly improved the AUC (0.79 [95% CI, 0.73–0.86]; *P* = 0.04). Likewise, the addition of uPRO-C6/Cr to the baseline model significantly improved the AUC to 0.80 (95% CI, 0.74–0.86; *P* = 0.002). A model that contained all variables from the baseline model plus uPRO-C3/Cr was not further improved when uPRO-C6/Cr was also added (*P* = 0.9; data not shown).

**Figure 2:**
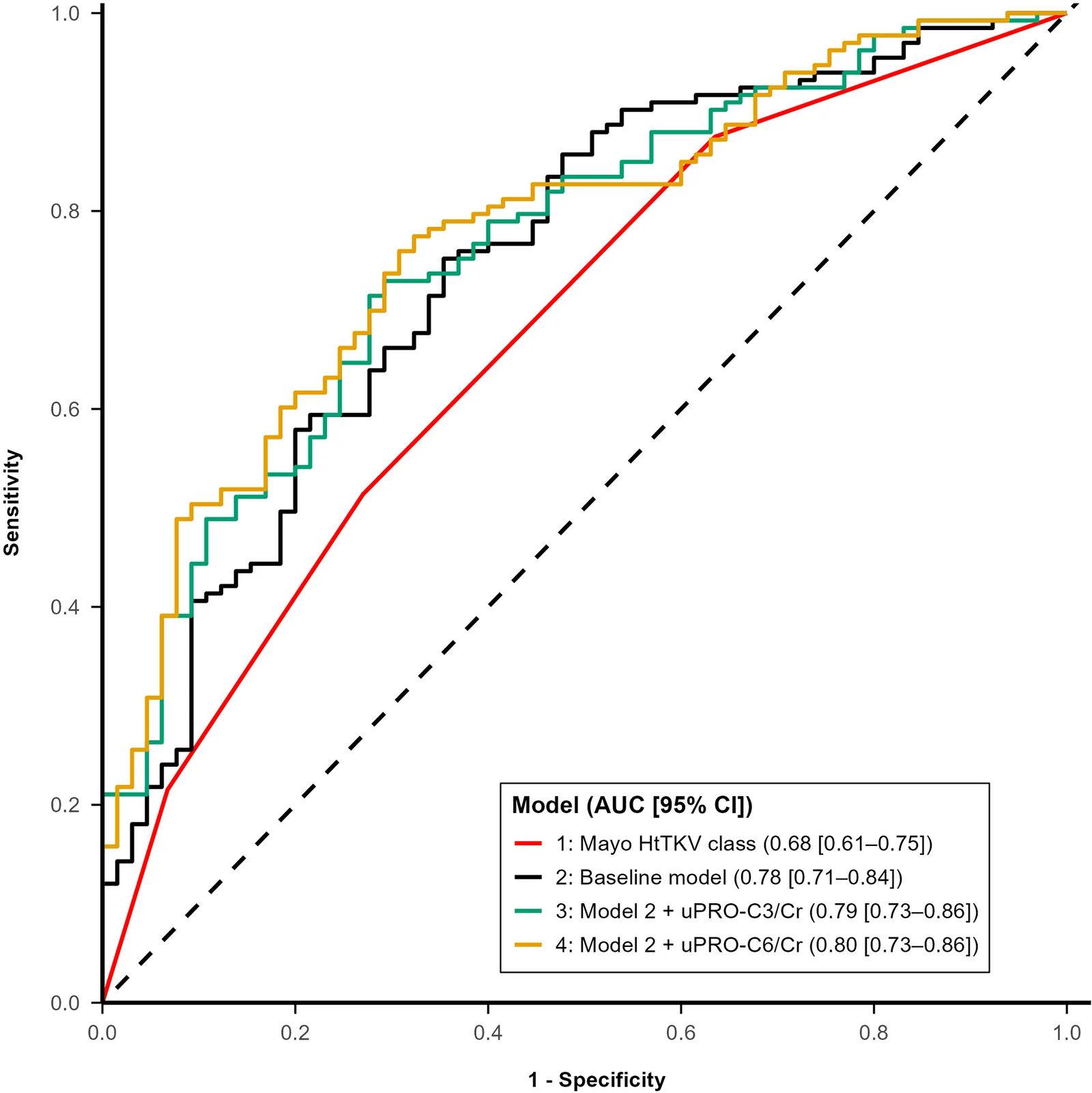
ROC curves of logistic regression models that predict the development of rapid disease progression (eGFR loss ≥ 3.0 ml/min per 1.73 m^2^ per year) for patients with available biomarker data. Baseline model refers to logistic regression models that include sex, baseline age, baseline eGFR, MIC, and *PKD* pathogenic variant type. When added to the baseline model, both uPRO-C3/Cr and uPRO-C6/Cr significantly improved the AUC. AUC, area under the curve; eGFR, estimated glomerular filtration rate; ROC, receiver operating characteristic.

To enable comparison to biomarkers previously associated with disease progression in ADPKD, we also investigated the addition of uMCP-1/Cr to the baseline model in this cohort, which did not substantially improve the AUC (0.78 [95% CI, 0.71–0.85]).

The results from net benefit analysis are presented in Supplementary Figure S2. In line with the modest increases in AUC, for both uPRO-C3/Cr and uPRO-C6/Cr, the added net benefit was not statistically significant for any decision threshold (respective averages across the range of decision thresholds: 0.0% [95% CI, −1.6 to 2.7] and 0.2% [95% CI, −2.2 to 3.0]). For uMCP-1/Cr, findings were similar (mean average across decision thresholds: 0.1% [95% CI, −1.6 to 2.7]).

### Kidney Survival Analysis

A total of 87 patients (28.8%) reached the composite endpoint of 30% eGFR decrease from baseline or kidney failure. Most of the patients who reached this composite outcome developed a 30% eGFR decline from baseline (*n* = 82). Overall, 5 patients reached an eGFR < 15 ml/min per 1.73 m^2^ and 4 patients received a preemptive kidney transplant.

Figure 3 shows the Kaplan-Meier estimates for the composite kidney outcome according to tertiles of baseline uPRO-C6/Cr, which predicted kidney survival (log rank *P* < 0.001). However, the log rank test of Kaplan-Meier estimates according to tertiles of uPRO-C3/Cr was not statistically significant (log rank *P* = 0.1, data not shown). The results of Cox regression survival analysis are displayed in Table 2. After adjustment for covariates, uPRO-C3/Cr and uPRO-C6/Cr remained independent predictors of the composite kidney endpoint (adjusted hazard ratio, 1.45 [95% CI, 1.15–1.83], *P* = 0.001, and 1.38 [95% CI, 1.12–1.72], *P* = 0.003, respectively). Cox regression models with corrections for an increasing number of covariates are displayed in Supplementary Table S10. For the baseline model containing sex, baseline age, baseline eGFR, MIC, *PKD* pathogenic variant type, and treatment group, the median C-index after bootstrapping was 0.71 (95% CI, 0.65–0.77). The addition of uPRO-C3/Cr and uPRO-C6/Cr respectively increased the C-index to 0.74 (95% CI, 0.67–0.80) and 0.72 (95% CI, 0.66–0.79). The addition of uPRO-C3/Cr to the baseline model significantly improved the model fit (*P* < 0.001), as did the addition of uPRO-C6/Cr (*P* = 0.006).

**Figure 3:**
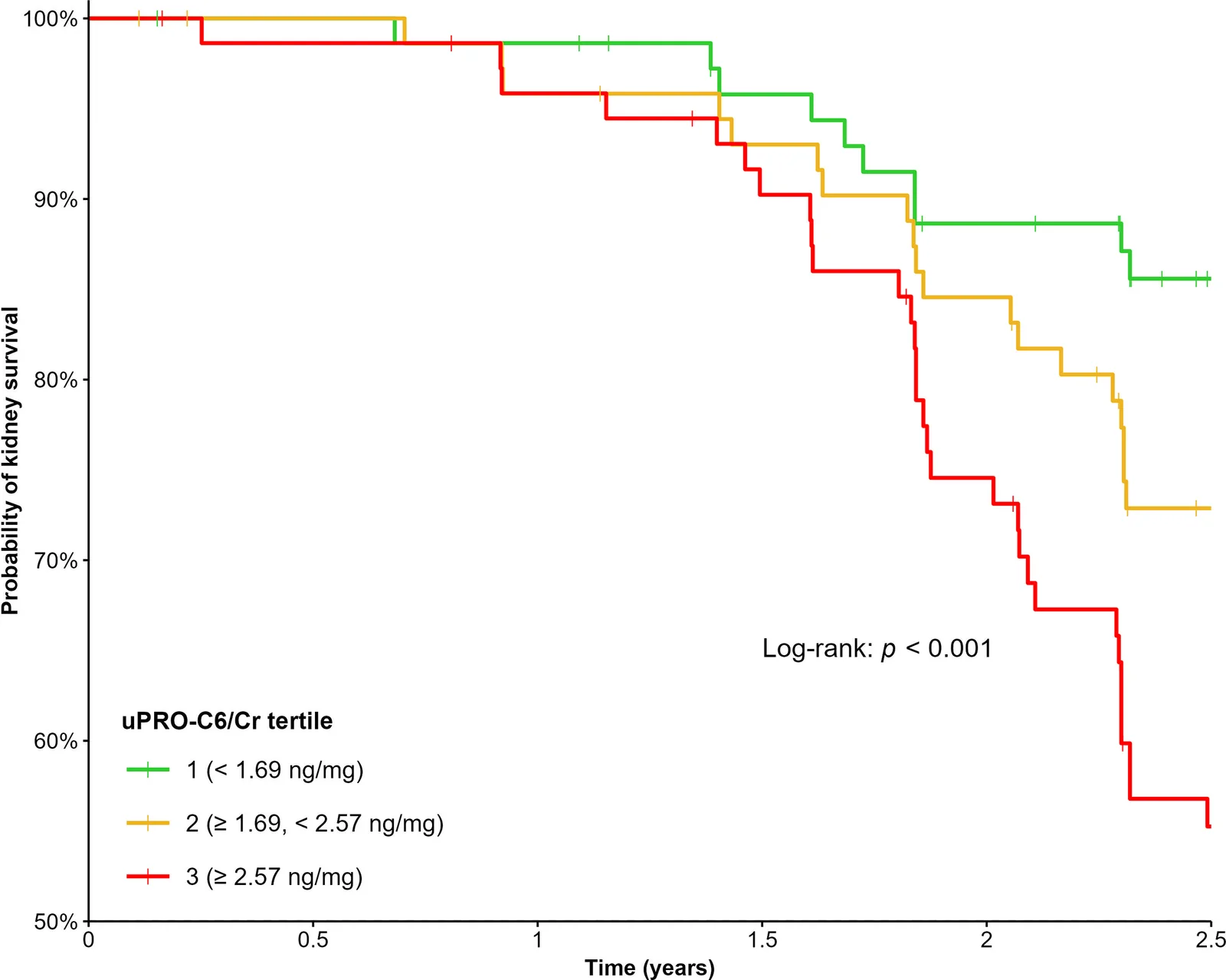
Kaplan-Meier survival analysis according to tertiles of uPRO-C6/Cr for the composite kidney endpoint of 30% eGFR decline from baseline or initiation of kidney replacement therapy (dialysis or kidney transplantation) or reaching eGFR <15 ml/min per 1.73 m^2^. eGFR, estimated glomerular filtration rate.

### Associations With HtTKV Growth Rate

The baseline biomarker association with HtTKV growth rate is displayed in Table 2. After correction for covariates, uPRO-C3/Cr was significantly associated with HtTKV growth rate (standardized β, 0.75 [95% CI, 0.06, 1.45]; *P* = 0.04). Linear mixed models for the effect on HtTKV growth rate corrected for an increasing number of covariates are displayed in Supplementary Table S11.

### Longitudinal Biomarker Slopes Versus eGFR Slope and HtTKV Growth Rate

The results of biomarker measurements at baseline, week12, 120, and 132 across patients with available data for all time points are displayed in Supplementary Table S6. No significant differences between the treatment groups were observed for any of the biomarkers at any timepoint. Contour and scatter plots of the change in biomarkers over time versus eGFR slope and HtTKV growth rate are displayed in Supplementary Figures S3 and S4 (Supplementary Methods). The correlations between biomarker slope and eGFR slope were strongest for sPRO-C6 (−0.68 [95% CI, −0.83 to −0.53]) and uC3M/Cr (0.83 [95% CI, 0.64–0.95]). Changes in uC3M/Cr were most strongly correlated with changes in HtTKV growth rate (−0.50 [95% CI, −0.78 to −0.16]).

### Sensitivity Analyses

Findings for urinary biomarkers using 24-hour urinary excretion instead of creatinine-corrected values were largely similar (Supplementary Table S12). Repeating the analyses with eGFR_cys_ values yielded similar results for the association of baseline uPRO-C6/Cr with eGFR slope and the composite kidney endpoint, but not for rapid disease progression (Supplementary Table S13). Models incorporating uACR alongside uPRO-C3/Cr and uPRO-C6/Cr are presented in Supplementary Tables S14 and S15, respectively. The association of uPRO-C3/Cr and uPRO-C6/Cr with kidney outcomes generally remained significant even after adjusting for uACR. Similarly, the associations remained significant after adjusting for uMCP-1/Cr (Supplementary Tables S16 and S17). Sensitivity analyses using imputation for missing data produced consistent results (Supplementary Table S18). The ratio of PRO-C3 or PRO-C6 to C3M in serum and urine (reflecting the balance between fibrogenesis and collagen breakdown) produced similar results to when PRO-C3 and PRO-C6 were analyzed individually (results not shown).

Subgroup analyses were performed by repeating the primary analyses in the 107 patients with MIC 1C. Disease progression parameters for this subgroup are presented in Supplementary Table S19. Standardized estimates were generally directionally consistent with those observed in the overall cohort (Supplementary Table S20), particularly for uPRO-C6/Cr, although most associations did not reach statistical significance, potentially owing to limited statistical power.

## Discussion

In this exploratory study, biomarkers reflecting fibrogenesis showed an association with disease severity and progression in patients with ADPKD. The biomarkers were associated with eGFR cross-sectionally after correction for covariates. In addition, baseline levels of uPRO-C3/Cr and uPRO-C6/Cr were independently associated with disease progression across various clinically relevant kidney endpoints, including eGFR slope, development of rapidly progressive disease, and the composite kidney endpoint. Baseline uPRO-C3/Cr levels were also associated with HtTKV growth rate. These results provide clinical evidence that ECM remodeling and fibrosis play a role in ADPKD disease progression.

uC3M/Cr and sC3M showed cross-sectional associations with baseline eGFR, but with opposite estimates. High sC3M levels were linked to lower eGFR, whereas high uC3M/Cr levels were linked to higher eGFR. A decrease in uC3M/Cr over time was associated with a more rapid rate of eGFR decline. Previous studies in kidney transplant recipients also found that uC3M/Cr levels decreased with advancing CKD.^22^ Likewise, studies with patients with IgA nephropathy and patients with high-risk CKD showed that uC3M/Cr levels decrease as CKD progresses, whereas sC3M levels increase, consistent with our findings in patients with ADPKD.^23^^,^^24^ Although little is known about the renal handling of these peptides, which complicates interpretation, the decreasing uC3M/Cr levels with progressing kidney disease may reflect a loss of the ability to degrade the accumulated type III collagen fibers as the tissue gets more fibrotic and collagen becomes cross-linked.

The addition of uPRO-C3/Cr and uPRO-C6/Cr significantly improved logistic regression models that predicted rapid disease progression, after adjustment for covariates. However, combining uPRO-C3/Cr and uPRO-C6/Cr within the same model did not further increase the discriminatory performance, probably because uPRO-C3/Cr and uPRO-C6/Cr levels were highly correlated and represent similar pathophysiological processes. In addition, the improvements in AUC values were statistically significant but modest, and decision curve analysis did not indicate significant net benefit for urinary PRO-C3, PRO-C6, or MCP-1. Importantly, because we wanted to study the incremental value beyond established prognostic predictors, our baseline models already incorporated a comprehensive set of variables, including sex, age, baseline eGFR, MIC, and genotype. No currently available risk stratification approaches combine volumetric and genetic information, meaning that our baseline models already contained substantial prognostic information. In this context, the incremental increase in AUC achievable by adding a single biomarker is expected to be limited. This is also illustrated by our comparison to urinary MCP-1, known from several studies to be associated with rapid disease progression in ADPKD,^20^^,^^25^ which did not significantly improve AUC when added to the baseline model. Furthermore, patients in DIPAK-1 were already selected for a relatively rapid rate of disease progression based on eGFR criteria, which reduces outcome heterogeneity and makes prediction modeling more challenging. Despite these considerations, we showed an independent association between ECM biomarkers and disease progression. Although the incremental improvement in discrimination beyond established prognostic models was minimal, these findings support a role for ECM remodeling in ADPKD progression. As such, the primary value of these biomarkers may lie in providing mechanistic insight into disease biology and potentially identifying patients with active fibrotic remodeling who could be considered for enrichment in future clinical trials targeting disease progression. Further studies in broader ADPKD populations are needed to better define their role alongside established prognostic factors.

According to our analyses, the association with kidney function endpoints was generally stronger for uPRO-C6/Cr than for uPRO-C3/Cr. The reason might be that PRO-C6 reflects the formation of type VI collagen and endotrophin. Endotrophin is a multifunctional proinflammatory and profibrotic matrikine that is implicated in multiple renal and extrarenal diseases.^26^ Its chemoattractant properties lead to macrophage accumulation, which is known to play a pathophysiologic role in ADPKD.^27^ Therefore, it is possible that uPRO-C6/Cr is more strongly associated with kidney endpoints than uPRO-C3/Cr, because it relates to multiple pathophysiological processes in the polycystic kidney.^26^ On the other hand, baseline uPRO-C3/Cr, but not uPRO-C6/Cr, was associated with HtTKV growth rate, potentially reflecting that interstitial collagens, particularly types III and I, are the predominant structural components of the ECM in polycystic kidneys.^3^

In general, the relationships between biomarker levels and baseline HtTKV and HtTKV growth rate were less pronounced compared with their associations with eGFR-based outcomes. This difference may be attributed to the fact that kidney volume growth in ADPKD is largely driven by cyst expansion, rather than changes in the interstitial matrix.

ECM peptide fragments, including PRO-C3 and PRO-C6, have previously shown potential as markers of kidney fibrosis and disease progression in kidney disease, including CKD,^24^^,^^28^^,^^29^ IgA nephropathy,^23^ lupus nephritis,^30^ and patients undergoing kidney transplant.^22^ These findings suggest a shared histopathological fibrotic process that contributes to kidney function decline. Because of the nature of this study, it is difficult to determine whether ECM biomarkers only reflect disease severity or whether they play pathophysiological roles in disease progression in ADPKD. For risk prediction, a biomarker’s utility depends on its prognostic value, not its causality or disease specificity. Still, experimental evidence suggests that fibrosis contributes to disease progression in ADPKD, as multiple animal models have shown ECM and basement membrane abnormalities in the disease,^31^, ^32^, ^33^ and inhibition of MMPs (which drive ECM remodeling) can attenuate disease progression in animal models.^34^^,^^35^ In human ADPKD, a previous study found MMP-7 levels in urinary extracellular vesicles to predict disease progression in patients with ADPKD.^36^ Furthermore, our results demonstrate that the association between uPRO-C3/Cr and uPRO-C6/Cr and kidney endpoints was independent of MCP-1, suggesting it may also be independent of inflammation. Hence, these findings suggest that increased fibrogenesis plays an important role in disease progression in ADPKD. Although our results cannot establish causality, these previous findings, combined with the independent association of baseline biomarker levels with kidney endpoints in our study, suggest that the biomarkers likely reflect pathophysiological processes that contribute to disease progression in ADPKD.

Interestingly, the correlations between systemic and urinary levels of C3M, PRO-C3, and PRO-C6 were weak. This may reflect that circulating and urinary biomarkers represent distinct biological compartments, with serum levels potentially reflecting systemic ECM turnover and urinary levels more closely reflecting local renal ECM remodeling. These proteins may be differentially released from renal tissue, variably retained or sequestered within cystic structures, or subject to differing degrees of tubular handling, including reabsorption and degradation. Furthermore, the renal handling of ECM-derived fragments remains poorly understood. Some of these fragments are relatively small peptide sequences, potentially comprising as few as 10 amino acids, and their filtration, reabsorption, degradation, and excretion pathways have not been systematically characterized. As a result, urinary concentrations may not directly mirror circulating levels. Together, these factors may contribute to the weak associations observed between circulating and urinary levels of C3M, PRO-C3, and PRO-C6, although the relative contribution of each mechanism remains unclear.

The strengths of this study include frequent serum creatinine measurements, a well-characterized cohort, repeated biomarker measurements, and repeated analysis with cystatin C-based eGFR. Study limitations encompass the relatively short follow-up time and the lack of an external validation cohort. Urinary biomarker data were available for 222 of 302 patients (73.5%), introducing a potential risk of selection bias. Although measured baseline characteristics were similar between patients with and without available urine samples and sensitivity analyses using multiple imputation yielded consistent results, subtle or unmeasured clinical heterogeneity in the subset with missing urine samples cannot be excluded and may limit the generalizability of the urinary biomarker findings. An additional limitation is the relatively small size of the MIC 1C subgroup (*n* = 107), which represents a clinically important population in whom additional prognostic markers are particularly needed. Although the observed associations were generally directionally consistent with those in the overall cohort, limited statistical power may have contributed to the lack of statistical significance. Although we compared ECM biomarkers with MCP-1, we could not include a broader panel of tubular injury markers, such as kidney injury molecule-1 or epidermal growth factor. In addition, for this exploratory study, the cohort consisted of patients selected for a certain rate of disease progression (based on eGFR criteria), which limits the generalizability of our results. The large proportion of Caucasian patients in this study further limits the generalizability to other ethnicities. Additional research in unselected patients with ADPKD is required to investigate the utility of these biomarkers in the general ADPKD population. Beyond association with clinical outcomes, ECM biomarkers could potentially help identify patients with a more active fibrotic phenotype, enrich trial populations for individuals at higher risk of progression, or serve as complementary markers of biological treatment response.

In this study, biomarkers of tubulointerstitial remodeling and fibrosis were independently associated with disease progression in patients with ADPKD. Given the lack of noninvasive tools assessing fibrosis, these biomarkers could potentially be useful as prognostic biomarkers of disease progression in ADPKD or applicable in clinical trials assessing pharmacodynamic effects of novel antifibrotic treatments in patients with ADPKD.

## Disclosure

SSG, PF, FG, and MAK are full-time employees at Nordic Bioscience, a privately owned, small to medium-size enterprise partly focused on the development of biomarkers. None of the authors received fees, bonuses or other benefits for the work described in this article. FG and MAK hold stock in Nordic Bioscience. The patents for the ELISAs used in this work are owned by Nordic Bioscience. RTG reports having received grants for research or financial compensation for consultancy from: Abbvie, Galapagos, GSK, Ipsen, Mironid, Otsuka Pharmaceuticals and Sanofi-Genzyme. EM reports research funding from: Ipsen, Otsuka Pharmaceuticals, and Sanofi-Genzyme. All other authors report no competing financial interests relevant to the results presented in this article.

## Supplementary Material

Supplementary File (PDF) **Supplementary Methods. Supplementary References. Figure S1**. Pearson correlation matrix of ln-transformed biomarkers at baseline. **Figure S2**. Decision curve analysis showing the incremental net benefit of adding uPRO-C3/Cr, uPRO-C6/Cr and uMCP-1/Cr to a baseline clinical prediction model containing sex, age, baseline eGFR, MIC, *PKD* pathogenic variant type and treatment group. Net benefit is calculated across a range of decision thresholds (0.1–0.5). Solid lines represent mean net benefit improvement over the baseline model, with shaded areas indicating bootstrapped 95% confidence intervals. **Figure S3**. Contour plot of posterior samples of eGFR slope versus slope of sC3M, sPRO-C6, uC3M/Cr, uPRO-C3/Cr, uPRO-C6/Cr. Black dots: posterior means for individual patients. Reddot: Posterior mean across all patients. **Figure S4**. Contour plot of posterior samples of HtTKV growth rate versus slope of sC3M, sPRO-C6, uC3M/Cr, uPRO-C3/Cr, uPRO-C6/Cr. Black dots: posterior means for individual patients. Reddot: Posterior mean across all patients. **Table S1**. Biomarker assay specifications and characteristics. **Table S2**. Overview of kidney endpoints in the DIPAK-1 trial. **Table S3**. Baseline characteristics for patients with stratification for uPRO-C3/Cr tertiles. **Table S4**. Baseline characteristics for patients with stratification for uPRO-C6/Cr tertiles. **Table S5**. Baseline characteristics for patients with stratification for the availability of urinary biomarker data. **Table S6**. Summary of biomarker levels for both treatment groups at the available timepoints. **Table S7**. Effects of biomarker levels on baseline eGFR and baseline HtTKV. **Table S8**. Linear mixed effects models that include baseline biomarkers levels to predict eGFR slope. **Table S9**. Logistic regression models that include baseline biomarkers levels to predict rapid disease progression. **Table S10**. Cox regression models that include baseline biomarkers levels to predict the composite kidney outcome. **Table S11**. Linear mixed effects models that include baseline biomarkers levels to predict HtTKV growth rate. **Table S12**. Summary of predictive models using baseline 24-hour urinary biomarker excretions. **Table S13**. Summary of predictive models for eGFR_cys_ based outcomes. **Table S14**. Summary of predictive models with uPRO-C3/Cr that include urinary albumin/creatinine ratio. **Table S15**. Summary of predictive models with uPRO-C6/Cr that include urinary albumin/creatinine ratio. **Table S16**. Summary of predictive models with uPRO-C3/Cr that include urinary MCP-1/creatinine ratio. **Table S17**. Summary of predictive models with uPRO-C6/Cr that include urinary MCP-1/creatinine ratio. **Table S18**. Summary of predictive models with imputation for missing data. **Table S19**. Overview of kidney endpoints in the DIPAK-1 trial in 107 patients with Mayo Imaging Class 1C. **Table S20**. Summary of predictive models that include baseline biomarkers levels to predict kidney function endpoints in 107 patients with Mayo Imaging Class 1C. CONSORT Checklist.
