# Supplementary material for: Fibrogenesis Biomarkers and Clinical Disease Progression in ADPKD
Source: Kidney Int Rep. 2026 Sep 23;11(12):107102. doi: 10.1016/j.ekir.2026.107102 (PMC13733330; doi:10.1016/j.ekir.2026.107102)
Supplement: Supplementary file 1 [file mmc1.pdf]

Supplementary Material

Tables

1. Supplementary Table 1: Biomarker assay specifications and characteristics
2. Supplementary Table 2: Overview of kidney endpoints in the DIPAK-1 trial
3. Supplementary Table 3. Baseline characteristics for patients with stratification for uPRO-C3/Cr tertiles
4. Supplementary Table 4. Baseline characteristics for patients with stratification for uPRO-C6/Cr tertiles
5. Supplementary Table 5. Baseline characteristics for patients with stratification for the availability of urinary biomarker data
6. Supplementary Table 6: Summary of biomarker levels for both treatment groups at the available timepoints
7. Supplementary Table 7: Effects of biomarker levels on baseline eGFR and baseline HtTKV
8. Supplementary Table 8. Linear mixed effects models that include baseline biomarkers levels to predict eGFR slope
9. Supplementary Table 9. Logistic regression models that include baseline biomarkers levels to predict rapid disease progression
10. Supplementary Table 10. Cox regression models that include baseline biomarkers levels to predict the composite kidney outcome
11. Supplementary Table 11. Linear mixed effects models that include baseline biomarkers levels to predict HtTKV growth rate
12. Supplementary Table 12: Summary of predictive models using baseline 24-hour urinary biomarker excretions
13. Supplementary Table 13: Summary of predictive models for $eGFR_{cys}$ based outcomes
14. Supplementary Table 14. Summary of predictive models with uPRO-C3/Cr that include urinary albumin/creatinine ratio
15. Supplementary Table 15. Summary of predictive models with uPRO-C6/Cr that include urinary albumin/creatinine ratio
16. Supplementary Table 16. Summary of predictive models with uPRO-C3/Cr that include urinary MCP-1/creatinine ratio
17. Supplementary Table 17. Summary of predictive models with uPRO-C6/Cr that include urinary MCP-1/creatinine ratio
18. Supplementary Table 18. Summary of predictive models with imputation for missing data

19. Supplementary Table 19. Overview of kidney endpoints in the DIPAK-1 trial in 107 patients with Mayo Imaging Class 1C

20. Supplementary Table 20. Summary of predictive models that include baseline biomarkers levels to predict kidney function endpoints in 107 patients with Mayo Imaging Class 1C

Figures

21. Supplementary Figure 1: Pearson correlation matrix of ln-transformed biomarkers at baseline

22. Supplementary Figure 2: Decision curve analysis showing the incremental net benefit of adding uPRO-C3/Cr, uPRO-C6/Cr and uMCP-1/Cr to a baseline clinical prediction model containing sex, age, baseline eGFR, MIC, *PKD* pathogenic variant type and treatment group. Net benefit is calculated across a range of decision thresholds (0.1–0.5). Solid lines represent mean net benefit improvement over the baseline model, with shaded areas indicating bootstrapped 95% confidence intervals.

23. Supplementary Figure 3: Contour plot of posterior samples of eGFR slope versus slope of sC3M, sPRO-C6, uC3M/Cr, uPRO-C3/Cr, uPRO-C6/Cr. Black dots: posterior means for individual patients. Red dot: Posterior mean across all patients

24. Supplementary Figure 4: Contour plot of posterior samples of HtTKV growth rate versus slope of sC3M, sPRO-C6, uC3M/Cr, uPRO-C3/Cr, uPRO-C6/Cr. Black dots: posterior means for individual patients. Red dot: Posterior mean across all patients

Tables

Supplementary Table 1. Biomarker assay specifications and characteristics

Biomarker	Description	Measures	Serum (BL and FU)	Urine (BL and FU)	Reference
C3M	MMP-9 cleaved fragment of type III collagen	Interstitial matrix degradation	✓	✓	[13], [S1]
PRO-C3	ADAMTS-2 cleaved N-terminal pro-peptide of collagen type III	Interstitial matrix formation	Only BL	✓	[14]
PRO-C6	C-terminal pro-peptide of type VI collagen (α3 chain)	Interstitial matrix formation and endotrophin	✓	✓	[15]

BL, Baseline; FU, Follow up visits; ADAMTS, a disintegrin and metalloproteinase with thrombospondin motifs; BMP, bone morphogenetic protein; MMP, matrix metalloproteinase

Supplementary Table 2. Overview of kidney endpoints in the DIPAK-1 trial. Continuous data are displayed as mean ± SD.

Variable	Overall (N=302)	Standard care (n=151)	Lanreotide (n=151)
Follow-up duration, years	2.4 ± 0.4	2.4 ± 0.5	2.5 ± 0.4
Available eGFR measurements, n	9 [8, 13]	9 [8, 13]	9 [8, 12]
eGFR slope, ml/min/1.73m^2/year	-4.02 ± 3.64	-4.17 ± 3.92	-3.87 ± 3.33
Rapid disease progression, n (%)	190 (62.9)	102 (67.5)	88 (58.3)
30% eGFR loss or kidney failure, n (%)	87 (28.8)	43 (28.5)	44 (29.1)

eGFR: estimated glomerular filtration rate

Supplementary Table 3. Baseline characteristics for patients with stratification for uPRO-C3/Cr tertiles

**Variable**	Overall (N=302)	uPRO-C3/Cr tertile (ng/mg) <5.24 (n=74)	5.24–8.23 (n=74)	>8.23 (n=74)	*p*-value
Male sex	140 (46.4)	44 (59.5)	35 (47.3)	18 (24.3)	<0.001
Age at baseline, years	48.9 ± 7.3	48.4 ± 6.9	49.1 ± 7.3	48.6 ± 7.7	0.83
Ethnicity					0.02
-Asian	3 (1.0)	1 (1.4)	1 (1.4)	0 (0.0)	
-Caucasian	294 (97.4)	68 (91.9)	73 (98.6)	74 (100.0)	
-Other	5 (1.7)	5 (6.8)	0 (0.0)	0 (0.0)	
BMI, kg/m^2	27.0 ± 4.7	26.8 ± 4.3	27.6 ± 5.4	26.6 ± 4.1	0.39
SBP, mmHg	132.9 ± 13.2	131.4 ± 12.3	134.3 ± 13.9	135.3 ± 12.4	0.17
Mayo Imaging Class					0.06
-1A/1B	49 (16.2)	12 (16.2)	12 (16.2)	11 (14.9)	
-1C	107 (35.4)	19 (25.7)	29 (39.2)	31 (41.9)	
-1D/1E	129 (42.7)	41 (55.4)	27 (36.5)	26 (35.1)	
-Atypical	11 (3.6)	1 (1.4)	3 (4.1)	6 (8.1)	
-Not available	6 (2.0)	1 (1.4)	3 (4.1)	0 (0.0)	
Pathogenic variant type					0.29
-*PKD2*	58 (19.2)	20 (27.0)	11 (14.9)	11 (14.9)	
-*PKD1* non-truncating	73 (24.2)	20 (27.0)	19 (25.7)	16 (21.6)	
-*PKD1* truncating	139 (46.0)	30 (40.5)	37 (50.0)	38 (51.4)	
-Not available	32 (10.6)	4 (5.4)	7 (9.5)	9 (12.2)	
eGFR, $ml/min/1.73m^2$	51.3 ± 11.5	52.3 ± 12.7	51.7 ± 11.7	49.1 ± 11.3	0.23

Data are given as number (with percentage) or mean ± SD. BMI, body mass index; eGFR: estimated glomerular filtration rate; *PKD*: polycystic kidney disease, SBP: systolic blood pressure

Supplementary Table 4. Baseline characteristics for patients with stratification for uPRO-C6/Cr tertiles

**Variable**	Overall (N=302)	uPRO-C6/Cr tertile (ng/mg) <1.69 (n=74)	1.69–2.56 (n=74)	>2.56 (n=74)	*p*-value
Male sex	140 (46.4)	43 (58.1)	32 (43.2)	22 (29.7)	0.002
Age at baseline, years	48.9 ± 7.3	48.9 ± 7.3	48.5 ± 6.7	48.7 ± 7.8	0.96
Ethnicity					0.17
-Asian	3 (1.0)	1 (1.4)	1 (1.4)	0 (0.0)	
-Caucasian	294 (97.4)	69 (93.2)	72 (97.3)	74 (100.0)	
-Other	5 (1.7)	4 (5.4)	1 (1.4)	0 (0.0)	
BMI, kg/m^2	27.0 ± 4.7	27.7 ± 5.2	26.6 ± 4.3	26.7 ± 4.3	0.24
SBP, mmHg	132.9 ± 13.2	132.2 ± 12.4	134.8 ± 13.5	134.1 ± 12.9	0.45
Mayo Imaging Class					0.42
-1A/1B	49 (16.2)	14 (18.9)	10 (13.5)	11 (14.9)	
-1C	107 (35.4)	23 (31.1)	24 (32.4)	32 (43.2)	
-1D/1E	129 (42.7)	34 (45.9)	33 (44.6)	27 (36.5)	
-Atypical	11 (3.6)	1 (1.4)	5 (6.8)	4 (5.4)	
-Not available	6 (2.0)	2 (2.7)	2 (2.7)	0 (0.0)	
Pathogenic variant type					0.47
-*PKD2*	58 (19.2)	20 (27.0)	11 (14.9)	11 (14.9)	
-*PKD1* non-truncating	73 (24.2)	16 (21.6)	21 (28.4)	18 (24.3)	
-*PKD1* truncating	139 (46.0)	31 (41.9)	35 (47.3)	39 (52.7)	
-Not available	32 (10.6)	7 (9.5)	7 (9.5)	6 (8.1)	
eGFR, ml/min/1.73m^2	51.3 ± 11.5	53.0 ± 12.1	52.7 ± 12.0	47.4 ± 10.9	0.005

Data are given as number (with percentage) or mean ± SD. BMI, body mass index; eGFR: estimated glomerular filtration rate; *PKD*: polycystic kidney disease, SBP: systolic blood pressure

Supplementary Table 5. Baseline characteristics for patients with stratification for the availability of urinary biomarker data

Variable	Overall (N=302)	Urinary biomarker data: Available (n=222)	Urinary biomarker data: Unavailable (n=80)	SMD	*p*-value
Male sex	140 (46.4)	97 (43.7)	43 (53.8)	0.20	0.16
Age at baseline, years	48.9 ± 7.3	48.7 ± 7.3	49.5 ± 7.5	0.10	0.42
Ethnicity				0.20	0.39
-Asian	3 (1.0)	2 (0.9)	1 (1.2)		
-Caucasian	294 (97.4)	215 (96.8)	79 (98.8)		
-Other	5 (1.7)	5 (2.3)	0 (0.0)		
BMI, kg/m^2	27.0 ± 4.7	27.0 ± 4.6	26.9 ± 5.0	0.02	0.89
SBP, mmHg	132.9 ± 13.2	133.7 ± 12.9	130.7 ± 13.9	0.22	0.09
Mayo Imaging Class				0.21	0.74
-1A/1B	49 (16.2)	35 (15.8)	14 (17.5)		
-1C	107 (35.4)	79 (35.6)	28 (35.0)		
-1D/1E	129 (42.7)	94 (42.3)	35 (43.8)		
-Atypical	11 (3.6)	10 (4.5)	1 (1.2)		
-Not available	6 (2.0)	4 (1.8)	2 (2.5)		
Pathogenic variant type				0.20	0.49
-*PKD2*	58 (19.2)	42 (18.9)	16 (20.0)		
-*PKD1* non-truncating	73 (24.2)	55 (24.8)	18 (22.5)		
-*PKD1* truncating	139 (46.0)	105 (47.3)	34 (42.5)		
-Not available	32 (10.6)	20 (9.0)	12 (15.0)		
eGFR, ml/min/1.73m^2	51.3 ± 11.5	51.1 ± 11.9	51.9 ± 10.2	0.08	0.58

Data are given as number (with percentage) or mean ± SD. BMI, body mass index; eGFR: estimated glomerular filtration rate; *PKD*: polycystic kidney disease, SBP: systolic blood pressure, SMD: standardized mean difference

Supplementary Table 6. Biomarker levels (median [IQR]) by treatment group at each timepoint (for patients with complete data across all timepoints).

**Biomarker**	**Timepoint (weeks)**	**Standard care**	**Lanreotide**	***p*-value**
**Serum**				
sC3M, ng/mL	0	15.8 [13.6, 18.9]	17.0 [14.3, 19.6]	0.30
	12	17.1 [15.2, 19.6]	17.5 [15.5, 19.6]	0.98
	120	17.7 [15.2, 19.4]	18.4 [15.6, 20.5]	0.24
	132	17.5 [15.4, 19.9]	17.5 [15.3, 20.9]	0.74
sPRO-C3, ng/mL	0	13.0 [10.9, 15.6]	12.5 [10.2, 16.0]	0.26
	12	-	-	-
	120	-	-	-
	132	-	-	-
sPRO-C6, ng/mL	0	11.3 [9.3, 13.7]	10.9 [9.4, 13.2]	0.72
	12	12.8 [10.8, 15.5]	11.6 [10.2, 14.5]	0.07
	120	15.1 [12.4, 18.8]	14.8 [11.8, 18.1]	0.43
	132	15.5 [12.7, 18.9]	16.3 [13.2, 20.1]	0.35
**Urine**				
uC3M/Cr, ng/mg	0	42.2 [36.6, 49.1]	45.9 [36.4, 57.1]	0.27
	12	34.1 [28.5, 41.1]	37.1 [31.5, 41.6]	0.18
	120	35.6 [25.7, 44.6]	33.2 [28.1, 38.7]	0.58
	132	33.5 [25.1, 38.1]	30.7 [24.9, 42.6]	0.87
uPRO-C3/Cr, ng/mg	0	6.46 [4.83, 8.75]	7.92 [4.73, 11.47]	0.33
	12	9.82 [7.03, 18.37]	8.81 [6.28, 23.87]	0.68
	120	8.58 [5.38, 18.72]	7.66 [5.76, 12.80]	0.99
	132	7.20 [5.64, 12.04]	6.67 [5.40, 11.54]	0.77
uPRO-C6/Cr, ng/mg	0	2.13 [1.66, 2.58]	2.39 [1.75, 3.49]	0.43
	12	1.84 [1.32, 2.41]	2.09 [1.31, 2.67]	0.68
	120	1.65 [1.18, 2.88]	2.10 [1.41, 3.64]	0.33
	132	1.91 [1.60, 3.72]	2.36 [1.31, 5.09]	0.87

Supplementary Table 7. Effects of biomarker levels on baseline eGFR (ml/m/1.73m^2/year) and baseline HtTKV (ml/m), corrected for sex, baseline age, baseline eGFR (except when this was the dependent variable), MIC, *PKD* pathogenic variant type, and treatment group. One biomarker was included per model. Biomarkers were ln-transformed and z-score standardized.

	**Baseline eGFR**		**Baseline HtTKV**	
Variable	St. β [95% CI]	*p*-value	St. β [95% CI]	*p*-value
**Serum**				
sC3M	-1.68 [-3.16, -0.20]	0.03	-25.9 [-90.0, 38.3]	0.43
sPRO-C3	-2.45 [-3.73, -1.17]	<0.001	-4.4 [-61.9, 53.1]	0.88
sPRO-C6	-6.25 [-7.42, -5.07]	<0.001	1.1 [-70.4, 72.4]	0.98
**Urine**				
uC3M/Cr	4.76 [3.16, 6.35]	<0.001	-49.3 [-128.0, 29.4]	0.22
uPRO-C3/Cr	-1.55 [-3.19, 0.10]	0.07	-15.3 [-85.3, 54.8]	0.67
uPRO-C6/Cr	-2.54 [-4.13, -0.95]	0.002	35.5 [-34.3, 105.2]	0.32

Supplementary Table 8. Linear mixed effects models that include baseline biomarkers levels to predict eGFR slope. Models are corrected for an increasing number of covariates. Model 4 is the full model that is also included in Table 2. One biomarker was included per model. Biomarkers were ln-transformed and z-score standardized.

	**Model 1 (Sex, age, treatment group)**		**Model 2 (Model 1 + baseline eGFR)**		**Model 3 (Model 2 + MIC)**		**Model 4 (Model 3 + pathogenic variant)**	
Variable	St. β [95% CI]	p-value	St. β [95% CI]	p-value	St. β [95% CI]	p-value	St. β [95% CI]	*p*-value
**Serum**								
sC3M	-0.35 [-0.66, -0.04]	0.03	-0.31 [-0.62, -0.00]	0.051	-0.33 [-0.64, -0.01]	0.04	-0.35 [-0.68, -0.01]	0.04
sPRO-C3	0.13 [-0.14, 0.41]	0.35	0.22 [-0.06, 0.50]	0.13	0.23 [-0.06, 0.51]	0.12	0.25 [-0.05, 0.55]	0.11
sPRO-C6	-0.33 [-0.61, -0.05]	0.02	-0.23 [-0.55, 0.10]	0.18	-0.24 [-0.56, 0.09]	0.16	-0.25 [-0.63, 0.14]	0.21
**Urine**								
uC3M/Cr	0.02 [-0.33, 0.38]	0.91	-0.12 [-0.51, 0.27]	0.54	-0.03 [-0.43, 0.36]	0.87	0.12 [-0.29, 0.53]	0.57
uPRO-C3/Cr	-0.57 [-0.90, -0.24]	0.001	-0.56 [-0.89, -0.22]	0.001	-0.55 [-0.89, -0.20]	0.002	-0.56 [-0.92, -0.21]	0.002
uPRO-C6/Cr	-0.80 [-1.11, -0.48]	<0.001	-0.79 [-1.12, -0.47]	<0.001	-0.79 [-1.11, -0.46]	<0.001	-0.74 [-1.08, -0.39]	<0.001

eGFR: estimated glomerular filtration rate; MIC: Mayo Imaging Class

Supplementary Table 9. Logistic regression models that include baseline biomarkers levels to predict rapid disease progression. Models are corrected for an increasing number of covariates. Model 4 is the full model that is also included in Table 2. One biomarker was included per model. Biomarkers were ln-transformed and z-score standardized.

	**Model 1 (Sex, age, treatment group)**		**Model 2 (Model 1 + baseline eGFR)**		**Model 3 (Model 2 + MIC)**		**Model 4 (Model 3 + pathogenic variant)**	
Variable	OR [95% CI]	p-value	OR [95% CI]	p-value	OR [95% CI]	p-value	OR [95% CI]	*p*-value
**Serum**								
sC3M	1.14 [0.87, 1.50]	0.33	1.07 [0.81, 1.42]	0.61	1.11 [0.82, 1.49]	0.49	1.01 [0.73, 1.39]	0.94
sPRO-C3	0.87 [0.68, 1.10]	0.24	0.79 [0.61, 1.01]	0.06	0.77 [0.59, 1.00]	0.052	0.77 [0.58, 1.02]	0.07
sPRO-C6	1.11 [0.87, 1.44]	0.41	0.90 [0.67, 1.21]	0.47	0.92 [0.68, 1.24]	0.58	0.84 [0.60, 1.19]	0.34
**Urine**								
uC3M/Cr	1.11 [0.82, 1.52]	0.50	1.35 [0.95, 1.93]	0.09	1.28 [0.89, 1.86]	0.19	1.14 [0.77, 1.69]	0.53
uPRO-C3/Cr	1.46 [1.07, 2.04]	0.02	1.41 [1.03, 1.98]	0.04	1.46 [1.05, 2.08]	0.03	1.46 [1.02, 2.14]	0.04
uPRO-C6/Cr	1.86 [1.31, 2.78]	0.001	1.79 [1.25, 2.69]	0.003	1.88 [1.29, 2.86]	0.002	1.92 [1.25, 3.13]	0.005

eGFR: estimated glomerular filtration rate; MIC: Mayo Imaging Class; OR: odds ratio

Supplementary Table 10. Cox regression models that include baseline biomarkers levels to predict the composite kidney endpoint. Models are corrected for an increasing number of covariates. Model 4 is the full model that is also included in Table 2. One biomarker was included per model. Biomarkers were ln-transformed and z-score standardized.

	**Model 1 (Sex, age, treatment group)**		**Model 2 (Model 1 + baseline eGFR)**		**Model 3 (Model 2 + MIC)**		**Model 4 (Model 3 + pathogenic variant)**	
Variable	HR [95% CI]	p-value	HR [95% CI]	p-value	HR [95% CI]	p-value	HR [95% CI]	*p*-value
**Serum**								
sC3M	1.25 [1.00, 1.57]	0.052	1.17 [0.94, 1.46]	0.15	1.18 [0.95, 1.47]	0.14	1.20 [0.95, 1.51]	0.14
sPRO-C3	1.07 [0.87, 1.31]	0.53	0.92 [0.74, 1.14]	0.44	0.91 [0.73, 1.12]	0.36	0.95 [0.76, 1.18]	0.64
sPRO-C6	1.41 [1.18, 1.67]	<0.001	1.10 [0.87, 1.39]	0.44	1.08 [0.84, 1.39]	0.53	1.18 [0.88, 1.59]	0.26
**Urine**								
uC3M/Cr	0.69 [0.53, 0.90]	0.006	0.94 [0.70, 1.27]	0.70	0.86 [0.62, 1.18]	0.34	0.78 [0.57, 1.08]	0.14
uPRO-C3/Cr	1.54 [1.23, 1.93]	<0.001	1.46 [1.17, 1.83]	0.001	1.49 [1.18, 1.87]	0.001	1.45 [1.15, 1.83]	0.001
uPRO-C6/Cr	1.62 [1.33, 1.97]	<0.001	1.43 [1.17, 1.74]	<0.001	1.46 [1.18, 1.80]	<0.001	1.38 [1.12, 1.72]	0.003

eGFR: estimated glomerular filtration rate; HR: hazard ratio; MIC: Mayo Imaging Class

Supplementary Table 11. Linear mixed effects models that include baseline biomarkers levels to predict HtTKV growth rate (in %/year). Models are corrected for an increasing number of covariates. Model 4 is the full model that is also included in Table 2. One biomarker was included per model. Biomarkers were ln-transformed and z-score standardized.

	**Model 1 (Sex, age, treatment group)**		**Model 2 (Model 1 + baseline eGFR)**		**Model 3 (Model 2 + MIC)**		**Model 4 (Model 3 + pathogenic variant)**	
Variable	St. β [95% CI]	p-value	St. β [95% CI]	p-value	St. β [95% CI]	p-value	St. β [95% CI]	*p*-value
**Serum**								
sC3M	0.18 [-0.42, 0.78]	0.56	0.08 [-0.52, 0.69]	0.79	0.06 [-0.55, 0.67]	0.86	0.25 [-0.39, 0.88]	0.45
sPRO-C3	0.10 [-0.43, 0.64]	0.71	-0.04 [-0.58, 0.51]	0.90	-0.05 [-0.60, 0.50]	0.87	0.07 [-0.51, 0.65]	0.81
sPRO-C6	0.53 [-0.00, 1.07]	0.053	0.28 [-0.34, 0.91]	0.38	0.28 [-0.35, 0.91]	0.39	0.31 [-0.41, 1.03]	0.41
**Urine**								
uC3M/Cr	-0.39 [-1.09, 0.32]	0.28	-0.22 [-0.98, 0.55]	0.57	-0.24 [-1.01, 0.54]	0.55	-0.18 [-0.98, 0.63]	0.66
uPRO-C3/Cr	0.71 [0.07, 1.35]	0.03	0.65 [0.01, 1.30]	0.047	0.63 [-0.03, 1.30]	0.06	0.75 [0.06, 1.45]	0.04
uPRO-C6/Cr	0.26 [-0.37, 0.89]	0.43	0.15 [-0.51, 0.80]	0.66	0.14 [-0.52, 0.80]	0.68	0.04 [-0.66, 0.74]	0.91

eGFR: estimated glomerular filtration rate; HtTKV: height-adjusted total kidney volume; MIC: Mayo Imaging Class

Supplementary Table 12. Summary of predictive models that include the baseline 24-hour excretion of urinary biomarkers levels. All models were corrected for sex, baseline age, baseline eGFR (except when this was the dependent variable), MIC, *PKD* pathogenic variant type, and treatment group. One biomarker was included per model. Biomarkers were ln-transformed and z-score standardized.

	**eGFR slope**		**Rapidly progressive disease**		**Composite kidney endpoint**		**HtTKV growth rate**	
Variable	St. β [95% CI]	p-value	OR [95% CI]	p-value	HR [95% CI]	p-value	St. β [95% CI]	*p*-value
uC3M	0.04 [-0.34, 0.41]	0.86	1.02 [0.73, 1.44]	0.90	0.90 [0.67, 1.19]	0.45	0.36 [-0.36, 1.09]	0.33
uPRO-C3	-0.46 [-0.81, -0.10]	0.01	1.31 [0.93, 1.87]	0.13	1.37 [1.08, 1.75]	0.01	0.96 [0.28, 1.65]	0.007
uPRO-C6	-0.60 [-0.96, -0.23]	0.002	1.53 [1.05, 2.35]	0.04	1.32 [1.05, 1.65]	0.02	0.38 [-0.33, 1.10]	0.29

Supplementary Table 13. Summary of predictive models for $eGFR_{cys}$ based outcomes. All models were corrected for sex, baseline age, baseline $eGFR_{cys}$ (except when this was the dependent variable), MIC, *PKD* pathogenic variant type, and treatment group. One biomarker was included per model. Biomarkers were ln-transformed and z-score standardized.

	**Baseline $eGFR_{cys}$**		**$eGFR_{cys}$ slope**		**Rapidly progressive disease**		**Composite kidney endpoint**	
Variable	St. β [95% CI]	*p*-value	St. β [95% CI]	*p*-value	OR [95% CI]	*p*-value	HR [95% CI]	*p*-value
**Serum**								
sC3M	-2.04 [-3.90, -0.18]	0.03	-0.24 [-0.56, 0.09]	0.16	1.21 [0.89, 1.67]	0.23	1.13 [0.89, 1.42]	0.32
sPRO-C3	-4.69 [-6.26, -3.11]	<0.001	0.56 [0.27, 0.85]	<0.001	0.64 [0.47, 0.85]	0.003	0.82 [0.65, 1.02]	0.08
sPRO-C6	-8.90 [-10.31, -7.49]	<0.001	0.06 [-0.33, 0.44]	0.78	0.86 [0.60, 1.24]	0.42	1.12 [0.83, 1.52]	0.46
**Urine**								
uC3M/Cr	2.75 [0.56, 4.93]	0.014	0.32 [-0.03, 0.67]	0.07	0.82 [0.57, 1.16]	0.26	0.87 [0.66, 1.17]	0.36
uPRO-C3/Cr	-1.48 [-3.59, 0.63]	0.17	-0.51 [-0.83, -0.19]	0.002	1.25 [0.89, 1.80]	0.21	1.51 [1.20, 1.91]	<0.001
uPRO-C6/Cr	-2.44 [-4.49, -0.39]	0.02	-0.55 [-0.87, -0.23]	0.001	1.20 [0.86, 1.71]	0.29	1.53 [1.23, 1.90]	<0.001

Supplementary Table 14. Summary of predictive models with inclusion of uPRO-C3/Cr and urinary albumin/creatinine. Biomarkers were ln-transformed and z-score standardized.

	**eGFR slope**		**Rapidly progressive disease**		**Composite kidney endpoint**		**HtTKV growth rate**	
Variable	St. β [95% CI]	*p*-value	OR [95% CI]	*p*-value	HR [95% CI]	*p*-value	St. β [95% CI]	*p*-value
Male sex	-0.84 [-1.61, -0.07]	0.03	2.83 [1.26, 6.68]	0.01	1.58 [0.88, 2.82]	0.13	3.52 [1.86, 5.20]	<0.001
Age, years	0.05 [-0.01, 0.12]	0.13	0.98 [0.91, 1.04]	0.47	0.96 [0.91, 1.02]	0.18	-0.14 [-0.23, 0.00]	0.04
eGFR, ml/min/1.73m^2	0.01 [-0.02, 0.04]	0.51	0.99 [0.96, 1.02]	0.39	0.94 [0.92, 0.97]	<0.001	-0.06 [-0.23, 0.00]	0.07
MIC								
-1C	-0.68 [-1.69, 0.32]	0.18	1.69 [0.66, 4.39]	0.28	1.33 [0.51, 3.43]	0.56	0.64 [-1.37, 2.57]	0.54
-1D	-0.64 [-1.80, 0.53]	0.28	2.19 [0.73, 6.75]	0.16	1.40 [0.50, 3.96]	0.52	0.15 [-2.28, 2.57]	0.90
-1E	-0.58 [-2.04, 0.88]	0.44	3.17 [0.69, 16.56]	0.15	0.73 [0.21, 2.53]	0.62	1.12 [-1.83, 3.99]	0.45
Pathogenic variant								
-*PKD1* non-truncating	-0.95 [-1.99, 0.10]	0.08	3.75 [1.38, 10.73]	0.01	0.95 [0.38, 2.37]	0.92	-2.04 [-4.06, 0.00]	0.06
-*PKD1* truncating	-1.02 [-2.01, -0.04]	0.04	4.51 [1.77, 11.97]	0.002	1.06 [0.45, 2.48]	0.90	-2.56 [-4.50, -0.69]	0.01
Lanreotide treatment	0.05 [-0.64, 0.73]	0.90	1.57 [0.79, 3.18]	0.21	0.71 [0.41, 1.24]	0.23	-2.32 [-3.62, -0.92]	0.001
uPRO-C3/Cr	-0.46 [-0.83, -0.09]	0.02	1.32 [0.91, 1.95]	0.16	1.37 [1.07, 1.74]	0.01	0.77 [0.00, 1.63]	0.04
uACR	-0.44 [-0.83, -0.05]	0.03	1.53 [1.04, 2.31]	0.04	1.50 [1.12, 2.02]	0.007	-0.02 [-0.92, 0.69]	0.96

eGFR: estimated glomerular filtration rate; MIC: Mayo Imaging Class; uACR: urinary albumin/creatinine ratio
Reference categories are MIC 1A/1B/atypical, and *PKD2* pathogenic variants

Supplementary Table 15. Summary of predictive models with inclusion of uPRO-C6/Cr and urinary albumin/creatinine. Biomarkers were ln-transformed and z-score standardized.

	**eGFR slope**		**Rapidly progressive disease**		**Composite kidney endpoint**		**HtTKV growth rate**	
Variable	St. β [95% CI]	*p*-value	OR [95% CI]	*p*-value	HR [95% CI]	*p*-value	St. β [95% CI]	*p*-value
Male sex	-0.72 [-1.46, 0.03]	0.06	2.74 [1.22, 6.51]	0.02	1.34 [0.77, 2.34]	0.31	3.31 [1.63, 4.95]	<0.001
Age, years	0.06 [-0.00, 0.13]	0.07	0.97 [0.91, 1.04]	0.38	0.96 [0.91, 1.01]	0.13	-0.13 [-0.23, 0.00]	0.06
eGFR, ml/min/1.73m^2	0.00 [-0.03, 0.03]	0.86	0.99 [0.96, 1.02]	0.54	0.94 [0.92, 0.97]	<0.001	-0.07 [-0.23, 0.00]	0.04
MIC								
-1C	-0.70 [-1.69, 0.28]	0.16	1.69 [0.64, 4.53]	0.29	1.40 [0.54, 3.62]	0.49	0.65 [-1.37, 2.80]	0.53
-1D	-0.54 [-1.67, 0.59]	0.35	2.09 [0.68, 6.53]	0.20	1.25 [0.45, 3.50]	0.68	-0.30 [-2.50, 2.09]	0.80
-1E	-0.48 [-1.91, 0.94]	0.51	3.07 [0.65, 16.5]	0.17	0.66 [0.20, 2.26]	0.51	0.64 [-2.28, 3.51]	0.67
Pathogenic variant								
-*PKD1* non-truncating	-0.88 [-1.91, 0.15]	0.10	3.48 [1.27, 10.05]	0.02	1.02 [0.42, 2.51]	0.97	-2.02 [-4.06, 0.00]	0.06
-*PKD1* truncating	-0.92 [-1.89, 0.05]	0.07	4.33 [1.67, 11.75]	0.003	1.02 [0.44, 2.37]	0.97	-2.35 [-4.28, -0.46]	0.02
Lanreotide treatment	0.02 [-0.66, 0.69]	0.96	1.61 [0.80, 3.31]	0.19	0.75 [0.44, 1.29]	0.30	-2.15 [-3.62, -0.69]	0.003
uPRO-C6/Cr	-0.65 [-1.01, -0.30]	<0.001	1.74 [1.14, 2.83]	0.02	1.32 [1.05, 1.65]	0.02	-0.04 [-0.69, 0.69]	0.92
uACR	-0.43 [-0.81, -0.06]	0.03	1.50 [1.02, 2.26]	0.04	1.54 [1.15, 2.06]	0.004	0.29 [-0.46, 1.16]	0.48

eGFR: estimated glomerular filtration rate; MIC: Mayo Imaging Class; uACR: urinary albumin/creatinine ratio
Reference categories are MIC 1A/1B/atypical, and *PKD2* pathogenic variants

Supplementary Table 16. Summary of predictive models with inclusion of uPRO-C3/Cr and urinary MCP-1/Cr. Biomarkers were ln-transformed and z-score standardized.

	**eGFR slope**		**Rapidly progressive disease**		**Composite kidney endpoint**		**HtTKV growth rate**	
Variable	St. β [95% CI]	*p*-value	OR [95% CI]	*p*-value	HR [95% CI]	*p*-value	St. β [95% CI]	*p*-value
Male sex	-0.68 [-1.43, 0.08]	0.08	2.14 [1.00, 4.76]	0.054	1.48 [0.84, 2.60]	0.18	3.55 [2.09, 5.20]	<0.001
Age, years	0.05 [-0.02, 0.12]	0.16	0.98 [0.92, 1.05]	0.60	0.96 [0.91, 1.01]	0.10	-0.13 [-0.23, 0.00]	0.06
eGFR, ml/min/1.73m^2	0.01 [-0.02, 0.04]	0.48	0.98 [0.95, 1.02]	0.32	0.94 [0.92, 0.97]	<0.001	-0.06 [-0.23, 0.00]	0.06
MIC								
-1C	-0.78 [-1.79, 0.22]	0.13	2.06 [0.82, 5.28]	0.13	1.34 [0.52, 3.47]	0.54	0.65 [-1.37, 2.57]	0.53
-1D	-0.81 [-1.96, 0.35]	0.18	2.73 [0.94, 8.18]	0.07	1.67 [0.60, 4.64]	0.33	0.18 [-2.05, 2.57]	0.88
-1E	-0.77 [-2.22, 0.67]	0.30	4.41 [1.02, 22.06]	0.06	0.86 [0.24, 3.02]	0.81	0.86 [-2.05, 3.75]	0.56
Pathogenic variant								
-*PKD1* non-truncating	-1.05 [-2.09, -0.01]	0.050	3.78 [1.42, 10.61]	0.009	1.03 [0.42, 2.54]	0.94	-2.05 [-4.06, 0.00]	0.052
-*PKD1* truncating	-0.92 [-1.91, 0.06]	0.07	3.68 [1.48, 9.46]	0.006	0.91 [0.39, 2.11]	0.83	-2.75 [-4.72, -0.92]	0.006
Lanreotide treatment	0.09 [-0.60, 0.78]	0.80	1.45 [0.73, 2.88]	0.29	0.74 [0.43, 1.27]	0.27	-2.35 [-3.62, -0.92]	0.001
uPRO-C3/Cr	-0.52 [-0.87, -0.16]	0.005	1.41 [0.99, 2.08]	0.07	1.41 [1.12, 1.78]	0.003	0.69 [0.00, 1.39]	0.06
uMCP-1/Cr	-0.32 [-0.70, 0.07]	0.11	1.25 [0.87, 1.80]	0.22	1.25 [0.92, 1.72]	0.16	0.31 [-0.46, 1.16]	0.43

eGFR: estimated glomerular filtration rate; uMCP-1/Cr: urinary monocyte chemoattractant protein 1/creatinine ratio; MIC: Mayo Imaging Class
Reference categories are MIC 1A/1B/atypical, and *PKD2* pathogenic variants

Supplementary Table 17. Summary of predictive models with inclusion of uPRO-C6/Cr and urinary MCP-1/Cr. Biomarkers were ln-transformed and z-score standardized.

	**eGFR slope**		**Rapidly progressive disease**		**Composite kidney endpoint**		**HtTKV growth rate**	
Variable	St. β [95% CI]	*p*-value	OR [95% CI]	*p*-value	HR [95% CI]	*p*-value	St. β [95% CI]	*p*-value
Male sex	-0.54 [-1.26, 0.18]	0.14	2.05 [0.96, 4.53]	0.07	1.23 [0.72, 2.12]	0.45	3.21 [1.62, 4.71]	<0.001
Age, years	0.06 [-0.01, 0.13]	0.08	0.98 [0.91, 1.04]	0.48	0.95 [0.90, 1.01]	0.08	-0.12 [-0.23, 0.00]	0.08
eGFR, ml/min/1.73m^2	0.00 [-0.03, 0.03]	0.85	0.99 [0.96, 1.02]	0.46	0.95 [0.92, 0.97]	<0.001	-0.07 [-0.23, 0.00]	0.04
MIC								
-1C	-0.81 [-1.79, 0.18]	0.11	2.06 [0.80, 5.42]	0.14	1.40 [0.54, 3.62]	0.49	0.72 [-1.37, 2.80]	0.48
-1D	-0.68 [-1.81, 0.45]	0.24	2.53 [0.85, 7.72]	0.10	1.46 [0.53, 4.06]	0.47	-0.14 [-2.50, 2.09]	0.91
-1E	-0.66 [-2.07, 0.76]	0.37	4.21 [0.94, 21.75]	0.07	0.73 [0.21, 2.54]	0.62	0.59 [-2.28, 3.51]	0.68
Pathogenic variant								
-*PKD1* non-truncating	-0.97 [-1.99, 0.06]	0.07	3.48 [1.29, 9.90]	0.02	1.11 [0.46, 2.69]	0.82	-1.96 [-4.06, 0.00]	0.07
-*PKD1* truncating	-0.82 [-1.79, 0.15]	0.10	3.52 [1.38, 9.26]	0.009	0.88 [0.38, 2.05]	0.78	-2.56 [-4.50, -0.69]	0.01
Lanreotide treatment	0.05 [-0.62, 0.73]	0.88	1.48 [0.74, 2.99]	0.27	0.79 [0.47, 1.34]	0.38	-2.22 [-3.62, -0.92]	0.002
uPRO-C6/Cr	-0.70 [-1.05, -0.35]	<0.001	1.86 [1.22, 3.03]	0.008	1.38 [1.11, 1.73]	0.004	-0.03 [-0.69, 0.69]	0.94
uMCP-1/Cr	-0.31 [-0.69, 0.06]	0.10	1.25 [0.87, 1.80]	0.23	1.32 [0.96, 1.81]	0.09	0.45 [-0.23, 1.16]	0.26

eGFR: estimated glomerular filtration rate; uMCP-1/Cr: urinary monocyte chemoattractant protein 1/creatinine ratio; MIC: Mayo Imaging Class
Reference categories are MIC 1A/1B/atypical, and *PKD2* pathogenic variants

Supplementary Table 18. Summary of predictive models with imputation for missing data. All models were corrected for sex, baseline age, baseline eGFR (except when this was the dependent variable), MIC, *PKD* pathogenic variant type, and treatment group. One biomarker was included per model. Biomarkers were ln-transformed and z-score standardized.

	**eGFR slope**		**Rapidly progressive disease**		**Composite kidney endpoint**		**HtTKV growth rate**	
Variable	St. β [95% CI]	p-value	OR [95% CI]	p-value	HR [95% CI]	p-value	St. β [95% CI]	*p*-value
**Serum**								
sC3M	-0.16 [-0.40, 0.08]	0.19	1.14 [0.85, 1.52]	0.39	1.20 [0.94, 1.53]	0.15	-0.14 [-0.77, 0.50]	0.68
sPRO-C3	0.25 [-0.02, 0.53]	0.07	0.74 [0.57, 0.96]	0.03	0.90 [0.73, 1.12]	0.35	-0.03 [-0.63, 0.57]	0.93
sPRO-C6	-0.25 [-0.57, 0.07]	0.13	0.94 [0.69, 1.26]	0.66	1.11 [0.87, 1.42]	0.42	0.22 [-0.47, 0.90]	0.54
**Urine**								
uC3M/Cr	-0.03 [-0.35, 0.29]	0.86	1.31 [0.93, 1.83]	0.12	0.92 [0.69, 1.23]	0.59	0.08 [-0.46, 0.62]	0.78
uPRO-C3/Cr	-0.31 [-0.56, -0.05]	0.02	1.46 [1.00, 2.14]	0.06	1.49 [1.19, 1.87]	<0.001	0.38 [-0.32, 1.09]	0.29
uPRO-C6/Cr	-0.43 [-0.68, -0.18]	<0.001	1.68 [1.14, 2.47]	0.01	1.45 [1.17, 1.81]	0.003	-0.09 [-0.71, 0.53]	0.78

Supplementary Table 19. Overview of kidney endpoints in the DIPAK-1 trial in 107 patients with Mayo Imaging Class 1C. Continuous data are displayed as mean ± SD.

**Variable**	
eGFR slope, ml/min/1.73m^2/year	-4.15 ± 3.45
Rapid disease progression, n (%)	67 (62.6)
30% eGFR loss or kidney failure, n (%)	32 (29.9)

eGFR: estimated glomerular filtration rate

Supplementary Table 20. Summary of predictive models that include baseline biomarkers levels to predict kidney function endpoints in patients with Mayo Imaging Class 1C (n=107). Kidney endpoints are eGFR slope, development of rapidly progressive disease, reaching the composite kidney endpoint and HtTKV growth rate. All models were corrected for sex, baseline age, baseline eGFR, *PKD* pathogenic variant type, and treatment group. One biomarker was included per model. Biomarkers were ln-transformed and z-score standardized.

	**eGFR slope**		**Rapidly progressive disease**		**Composite kidney endpoint**		**HtTKV growth rate**	
Variable	St. β [95 CI]	p-value	OR [95 CI]	p-value	HR [95 CI]	p-value	St. β [95 CI]	*p*-value
**Serum**								
sC3M	-0.86 [-1.46, -0.25]	0.01	1.64 [0.91, 3.20]	0.12	1.56 [0.89, 2.75]	0.12	-0.24 [-1.14, 0.67]	0.60
sPRO-C3	0.45 [-0.06, 0.96]	0.09	0.68 [0.41, 1.08]	0.11	0.94 [0.61, 1.45]	0.79	-0.24 [-1.00, 0.52]	0.54
sPRO-C6	0.15 [-0.50, 0.80]	0.65	0.82 [0.46, 1.48]	0.51	0.96 [0.56, 1.62]	0.87	0.31 [-0.61, 1.23]	0.51
**Urine**								
uC3M/Cr	0.60 [-0.16, 1.37]	0.13	0.76 [0.37, 1.55]	0.44	0.53 [0.31, 0.92]	0.02	0.29 [-0.86, 1.45]	0.62
uPRO-C3/Cr	-0.07 [-0.67, 0.53]	0.82	1.18 [0.69, 2.11]	0.55	1.14 [0.78, 1.69]	0.50	-0.31 [-1.17, 0.56]	0.49
uPRO-C6/Cr	-0.69 [-1.43, 0.06]	0.08	1.91 [0.93, 4.30]	0.10	1.20 [0.72, 2.01]	0.49	-1.03 [-2.12, 0.07]	0.07

eGFR: estimated glomerular filtration rate; HtTKV: height-adjusted total kidney volume; OR: odds ratio; HR: hazard ratio

Figures

Supplementary Figure 1. Pearson correlation matrix of ln-transformed biomarkers at baseline. * $p < 0.05$, ** $p < 0.01$, *** $p < 0.001$

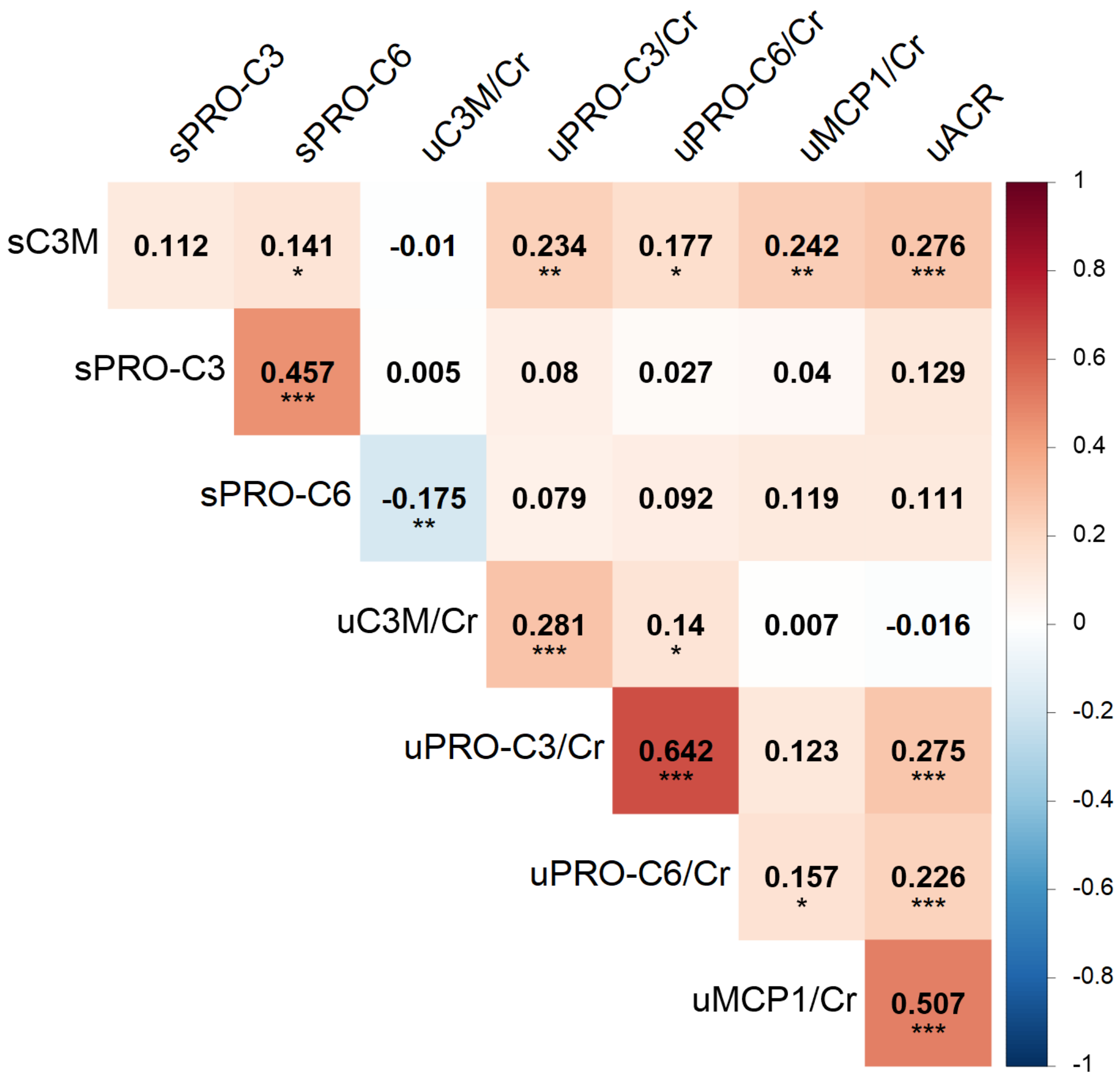

Supplementary Figure 2. Decision curve analysis showing the incremental net benefit of adding uPRO-C3/Cr, uPRO-C6/Cr and uMCP-1/Cr to a baseline clinical prediction model containing sex, age, baseline eGFR, MIC, *PKD* pathogenic variant type and treatment group. Net benefit is calculated across a range of decision thresholds (0.1–0.5). Solid lines represent mean net benefit improvement over the baseline model, with shaded areas indicating bootstrapped 95% confidence intervals.

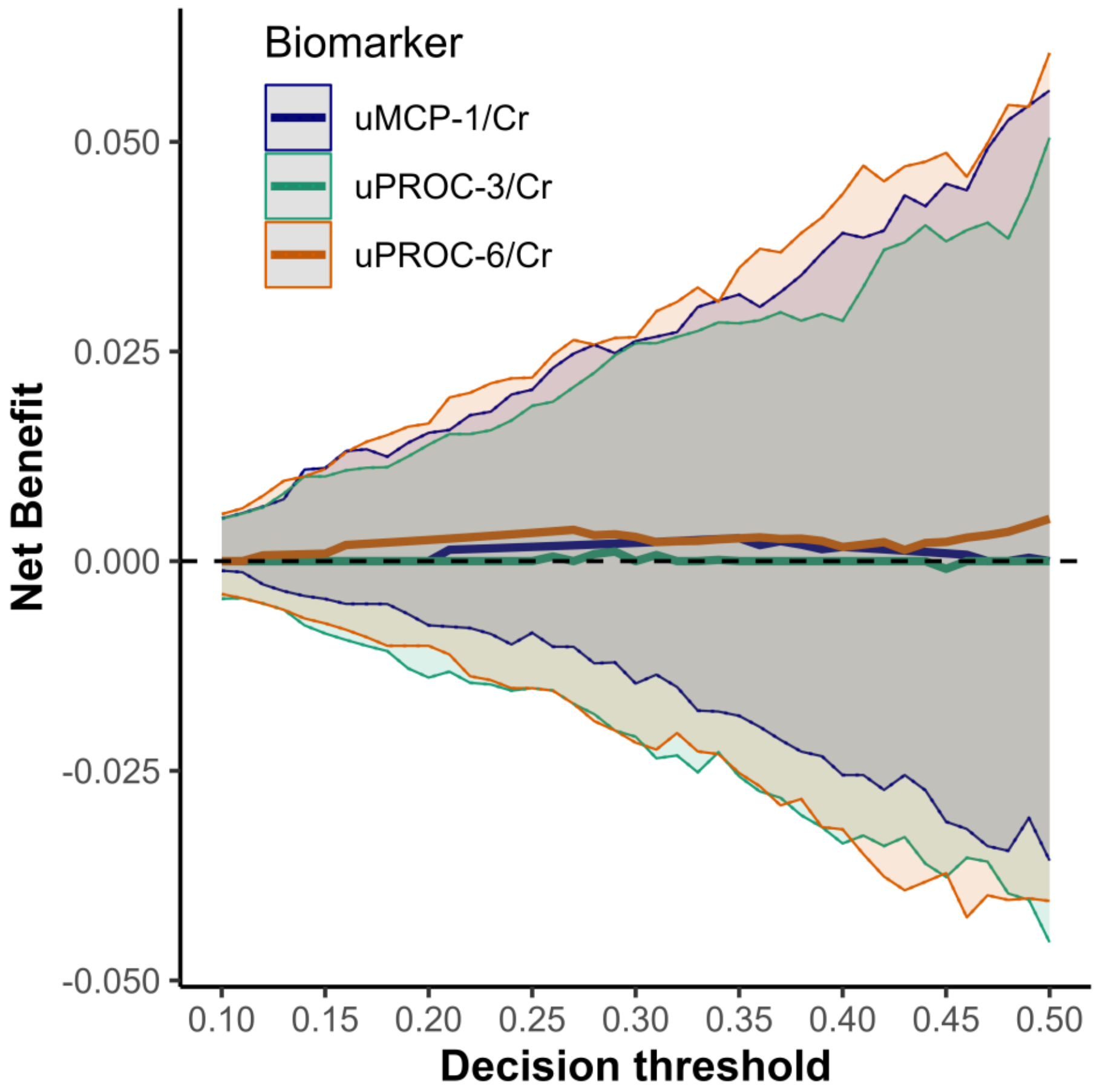

Supplementary Figure 3. Contour plot of posterior samples of slope in eGFR versus slope in sC3M, sPRO-C6, uC3M/Cr, uPRO-C3/Cr, uPRO-C6/Cr. Black dots: posterior means for individual patients. Red dot: Posterior mean across all patients

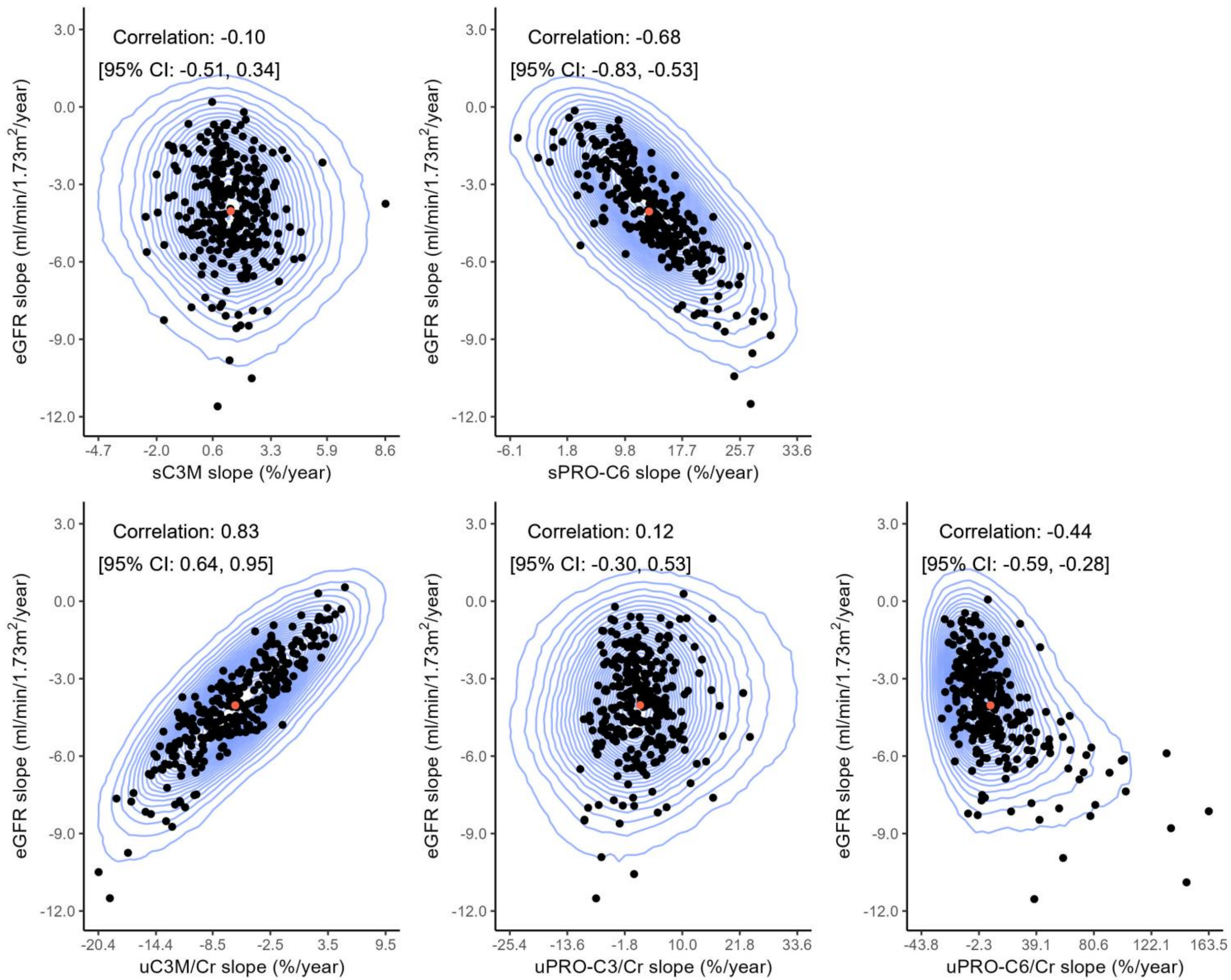

Supplementary Figure 4. Contour plot of posterior samples of HtTKV growth rate versus slope in sC3M, sPRO-C6, uC3M/Cr, uPRO-C3/Cr, uPRO-C6/Cr. Black dots: posterior means for individual patients. Red dot: Posterior mean across all patients

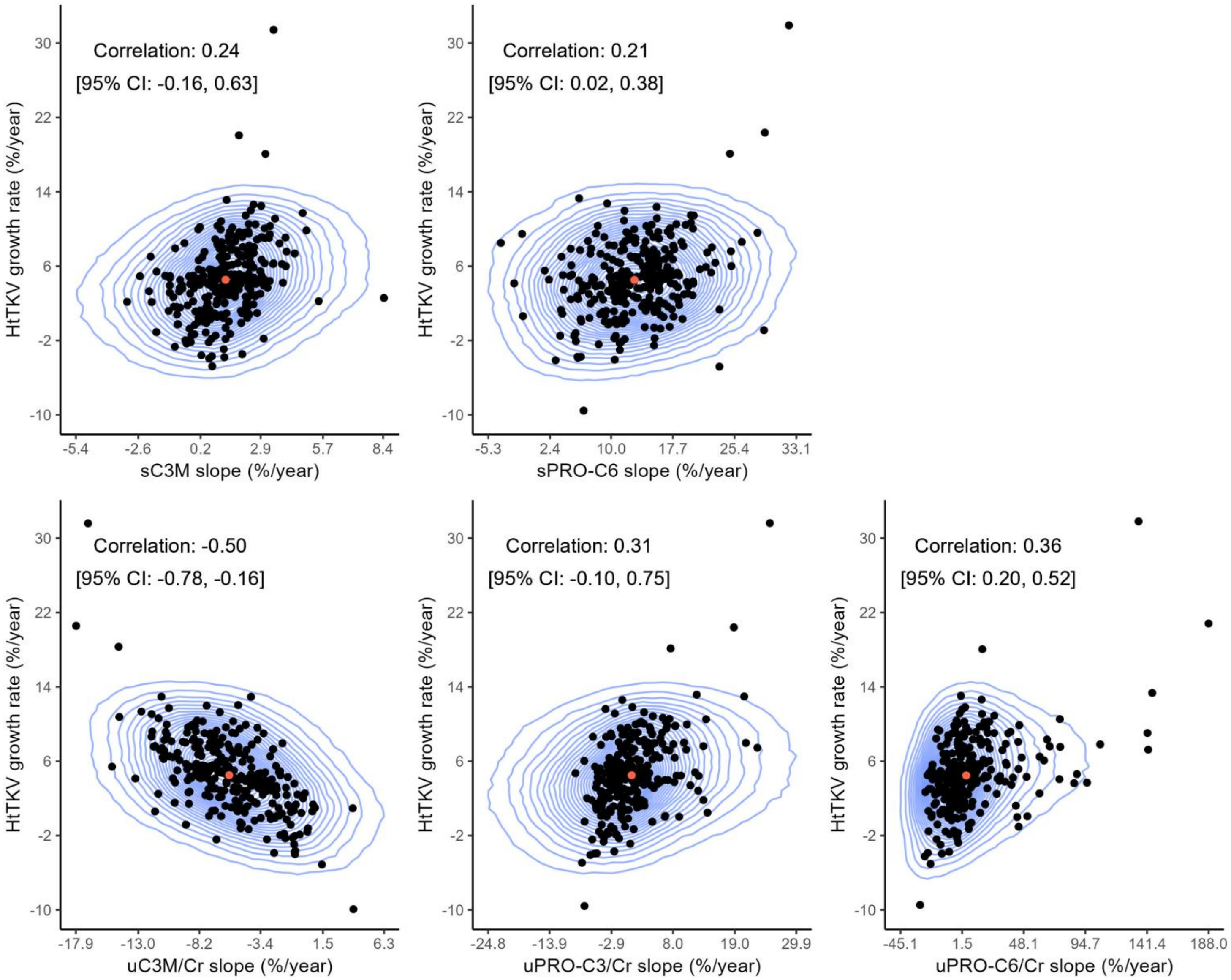

Supplementary Methods

For the correlation between longitudinal measurements bivariate random intercept random slope models were employed. The models explicitly include the covariance between random effects in the longitudinal models for the markers and the random effects in the longitudinal models for eGFR slope or HtTKV growth rate, allowing estimation of the population correlation between biomarker slopes and eGFR slope or HtTKV growth rate. The bivariate longitudinal models were specified within a Bayesian framework and fitted using the 'rstanarm' package[S2]. The software's weakly informative default priors were employed, and posterior summaries were based on 10,000 samples. From the posterior samples, the parameters representing the population average slopes and the parameters representing subject-specific deviations from the population average were combined to obtain posterior samples of subject-specific slopes. The posterior samples of subject-specific slopes were visualized using 2D contour plots.

Supplementary References

[S1] N. Barascuk *et al.*, "A novel assay for extracellular matrix remodeling associated with liver fibrosis: An enzyme-linked immunosorbent assay (ELISA) for a MMP-9 proteolytically revealed neo-epitope of type III collagen," *Clinical Biochemistry*, vol. 43, no. 10, pp. 899–904, 2010.

[S2] A. I. & B. S. Goodrich B, Gabry J, "rstanarm: Bayesian applied regression modeling via Stan." [Online]. Available: https://mc-stan.org/rstanarm

CONSORT 2025 checklist of information to include when reporting a randomised trial*

Section / Topic	No	CONSORT 2025 checklist item description	Reported on page no.
**Title and abstract**			
Title and structured abstract	1a	Identification as a randomised trial	7
	1b	Structured summary of the trial design, methods, results, and conclusions	2
**Open science**			
Trial registration	2	Name of trial registry, identifying number (with URL) and date of registration	7
Protocol and statistical analysis plan	3	Where the trial protocol and statistical analysis plan can be accessed	7-10
Data sharing	4	Where and how the individual de-identified participant data (including data dictionary), statistical code and any other materials can be accessed	22
Funding and conflicts of interest	5a	Sources of funding and other support (e.g., supply of drugs), and role of funders in the design, conduct, analysis and reporting of the trial	22
	5b	Financial and other conflicts of interest of the manuscript authors	22
**Introduction**			
Background and rationale	6	Scientific background and rationale	5-6
Objectives	7	Specific objectives related to benefits and harms	5-6
**Methods**			
Patient and public involvement	8	Details of patient or public involvement in the design, conduct and reporting of the trial	7-8
Trial design	9	Description of trial design including type of trial (e.g., parallel group, crossover), allocation ratio, and framework (e.g., superiority, equivalence, non-inferiority, exploratory)	7-8
Changes to trial protocol	10	Important changes to the trial after it commenced including any outcomes or analyses that were not prespecified, with reason	7-8
Trial setting	11	Settings (e.g., community, hospital) and locations (e.g., countries, sites) where the trial was conducted	7-8
Eligibility criteria	12a	Eligibility criteria for participants	7-8
	12b	If applicable, eligibility criteria for sites and for individuals delivering the interventions (e.g., surgeons, physiotherapists)	7-8

Intervention and comparator	13	Intervention and comparator with sufficient details to allow replication. If relevant, where additional materials describing the intervention and comparator (e.g., intervention manual) can be accessed	7-8
Outcomes	14	Pre-specified primary and secondary outcomes, including the specific measurement variable (e.g., systolic blood pressure), analysis metric (e.g., change from baseline, final value, time to event), method of aggregation (e.g., median, proportion), and time point for each outcome	9-10
Harms	15	How harms were defined and assessed (e.g., systematically, non-systematically)	-
Sample size	16a	How sample size was determined, including all assumptions supporting the sample size calculation	-
	16b	Explanation of any interim analyses and stopping guidelines	-
Randomisation:			
Sequence generation	17a	Who generated the random allocation sequence and the method used	-
	17b	Type of randomisation and details of any restriction (e.g., stratification, blocking and block size)	-
Allocation concealment mechanism	18	Mechanism used to implement the random allocation sequence (e.g., central computer/telephone; sequentially numbered, opaque, sealed containers), describing any steps to conceal the sequence until interventions were assigned	-
Implementation	19	Whether the personnel who enrolled and those who assigned participants to the interventions had access to the random allocation sequence	-
Blinding	20a	Who was blinded after assignment to interventions (e.g., participants, care providers, outcome assessors, data analysts)	9-10
	20b	If blinded, how blinding was achieved and description of the similarity of interventions	-
Statistical methods	21a	Statistical methods used to compare groups for primary and secondary outcomes, including harms	9-11
	21b	Definition of who is included in each analysis (e.g., all randomised participants), and in which group	9-11
	21c	How missing data were handled in the analysis	8-9, 11
	21d	Methods for any additional analyses (e.g., subgroup and sensitivity analyses), distinguishing prespecified from post-hoc	9-12
**Results**			
Participant flow, including flow diagram	22a	For each group, the numbers of participants who were randomly assigned, received intended intervention, and were analysed for the primary outcome	13-17
	22b	For each group, losses and exclusions after randomisation, together with reasons	-
Recruitment	23a	Dates defining the periods of recruitment and follow-up for outcomes of benefits and harms	9
	23b	If relevant, why the trial ended or was stopped	-

Intervention and comparator delivery	24a	Intervention and comparator as they were actually administered (e.g., where appropriate, who delivered the intervention/comparator, how participants adhered, whether they were delivered as intended [fidelity])	-
	24b	Concomitant care received during the trial for each group	-
Baseline data	25	A table showing baseline demographic and clinical characteristics for each group	28
Numbers analysed, outcomes and estimation	26	For each primary and secondary outcome, by group:<br>● the number of participants included in the analysis<br>● the number of participants with available data at the outcome time point<br>● result for each group, and the estimated effect size and its precision (such as 95% confidence interval)<br>● for binary outcomes, presentation of both absolute and relative effect size	9-12
Harms	27	All harms or unintended events in each group	-
Ancillary analyses	28	Any other analyses performed, including subgroup and sensitivity analyses, distinguishing pre-specified from post-hoc	11
**Discussion**			
Interpretation	29	Interpretation consistent with results, balancing benefits and harms, and considering other relevant evidence	18-20
Limitations	30	Trial limitations, addressing sources of potential bias, imprecision, generalisability, and, if relevant, multiplicity of analyses	21

*We strongly recommend reading this statement in conjunction with the CONSORT 2025 Explanation and Elaboration and/or the CONSORT 2025 Expanded Checklist for important clarifications on all the items. We also recommend reading relevant CONSORT extensions. See www.consort-spirit.org.

Citation: Hopewell S, Chan AW, Collins GS, Hróbjartsson A, Moher D, Schulz KF, et al. CONSORT 2025 Statement: updated guideline for reporting randomised trials. BMJ. 2025; 388:e081123. https://dx.doi.org/10.1136/bmj-2024-081123.

© 2025 Hopewell et al. This is an Open Access article distributed under the terms of the Creative Commons Attribution License (https://creativecommons.org/licenses/by/4.0/), which permits unrestricted use, distribution, and reproduction in any medium, provided the original work is properly cited.
